# Diallyl Disulfide: A Bioactive Garlic Compound with Anticancer Potential

**DOI:** 10.3389/fphar.2022.943967

**Published:** 2022-08-22

**Authors:** Saikat Mitra, Rajib Das, Talha Bin Emran, Rafiuddin Khan Labib, Noor-E-Tabassum, Fahadul Islam, Rohit Sharma, Islamudin Ahmad, Firzan Nainu, Kumarappan Chidambaram, Fahad A. Alhumaydhi, Deepak Chandran, Raffaele Capasso, Polrat Wilairatana

**Affiliations:** ^1^ Department of Pharmacy, Faculty of Pharmacy, University of Dhaka, Dhaka, Bangladesh; ^2^ Department of Pharmacy, BGC Trust University Bangladesh, Chittagong, Bangladesh; ^3^ Department of Pharmacy, Faculty of Allied Health Sciences, Daffodil International University, Dhaka, Bangladesh; ^4^ Department of Rasa Shastra and Bhaishajya Kalpana, Faculty of Ayurveda, Institute of Medical Sciences, Banaras Hindu University, Varanasi, India; ^5^ Department of Pharmaceutical Sciences, Faculty of Pharmacy, Mulawarman University, Samarinda, Indonesia; ^6^ Department of Pharmacy, Faculty of Pharmacy, Hasanuddin University, Makassar, Indonesia; ^7^ Department of Pharmacology and Toxicology, College of Pharmacy, King Khalid University, Abha, Saudi Arabia; ^8^ Department of Medical Laboratories, College of Applied Medical Sciences, Qassim University, Buraydah, Saudi Arabia; ^9^ Department of Veterinary Sciences and Animal Husbandry, Amrita School of Agricultural Sciences, Amrita Vishwa Vidyapeetham University, Coimbatore, India; ^10^ Department of Agricultural Sciences, University of Naples Federico II, Naples, Italy; ^11^ Department of Clinical Tropical Medicine, Faculty of Tropical Medicine, Mahidol University, Bangkok, Thailand

**Keywords:** oncology, garlic, diallyl disulfide, clinical, pre-clinical, Pharmacology, drug discovery

## Abstract

Cancer is a life-threatening disease caused by the uncontrolled division of cells, which culminates in a solid mass of cells known as a tumor or liquid cancer. It is the leading cause of mortality worldwide, and the number of cancer patients has been increasing at an alarming rate, with an estimated 20 million cases expected by 2030. Thus, the use of complementary or alternative therapeutic techniques that can help prevent cancer has been the subject of increased attention. Garlic, the most widely used plant medicinal product, exhibits a wide spectrum of biological activities, including antibacterial, hypo-lipidemic, antithrombotic, and anticancer effects. Diallyl disulfide (DADS) is a major organosulfur compound contained within garlic. Recently, several experimental studies have demonstrated that DADS exhibits anti-tumor activity against many types of tumor cells, including gynecological cancers (cervical cancer, ovarian cancer), hematological cancers (leukemia, lymphoma), lung cancer, neural cancer, skin cancer, prostate cancer, gastrointestinal tract and associated cancers (esophageal cancer, gastric cancer, colorectal cancer), hepatocellular cancer cell line, etc. The mechanisms behind the anticancer action of DADS include epithelial-mesenchymal transition (EMT), invasion, and migration. This article aims to review the available information regarding the anti-cancer potential of DADS, as well as summarize its mechanisms of action, bioavailability, and pharmacokinetics from published clinical and toxicity studies.

## 1 Introduction

Cancer is a severely detrimental health condition that is experienced by people across the world. The number of cancer-afflicted patients is rising rapidly and is estimated to be around 20 million by 2030 (Nussbaumer et al., 2011; Munari et al., 2014; Siegel et al., 2016; Society, 2016). Unbridled cellular growth leads to the modification of internal cellular or tissue materials as well as genetic instabilities, converting normal, healthy cells into malignant ones (Feller et al., 2013; Rauf et al., 2021). These genetic instabilities include mutations in the oncogenes, tumor suppressors, DNA repairing genes, and genes involved in the metabolism of cell growth (Dixon and Kopras, 2004). Cancer has several internal (hormonal abnormalities, genetic mutations, and the immune system) and external (smoking, cigarettes, drinking polluted water, food, radiation, chemicals, heavy metals, air, and infectious agents) causes (Bigby, 1988; Zhou et al., 2017; Mitra et al., 2022a). Thus, the discovery and development of phytochemical compounds that can be used as promising drugs for carcinoma treatment purposes have become a strong priority for researchers (Mitra et al., 2022e; Rahman et al., 2022).

Current therapeutic strategies for cancer treatment include surgery, radiotherapy, and chemotherapy, among others. However, patients undergoing these cancer preventative therapies experience intense stress; potentially leading to damaging, long-term health issues (Haberkorn, 2007; Moding et al., 2013; Mitra et al., 2022d). Several studies have shown that many medicinal plant species can be used to prevent or cure cancer (Mitra et al., 2021, 2022c; Islam et al., 2022). Anticancer effects have been observed in many plant species, especially in plants that have been utilized in traditional herbal treatments used in developing countries (Greenwell and Rahman, 2015). It is estimated that dietary modifications could prevent nearly one-third of current cancer cases (Brennan et al., 2010; Rastegari and Rafieian-Kopaei, 2016; Rauf et al., 2022).

Garlic, the most widely used plant in medicinal products, has several pharmacological properties, such as antibacterial, hypolipidemic, antithrombotic, and anticancer effects (Lai et al., 2013; Mikaili et al., 2013). Garlic oil is soluble in both oil and water, and it has long been considered to be both a dietary supplement and an anticancer agent (Omar and Al-Wabel, 2010). Experimental animal studies have shown that specific sulfur-containing compounds can chemically suppress carcinogenesis in different organs (Kim et al., 2014). Diallyl disulfide (DADS), a compound composed of two allyl groups connected by two sulfur atoms, is a vital organosulfur compound found in garlic (De Greef et al., 2021; He et al., 2021; Song et al., 2021). DADS is produced during the decomposition of allicin, and there have been many detailed studies regarding its medicinal applications (Liang et al., 2015). DADS, diallyl tetrasulfide, and diallyl trisulfide are the key elements of distilled garlic oil. DADS is a yellow liquid that has a distinct garlic odor and is not soluble in water. Allicin is released when garlic or other plants belonging to the family Alliaceae are crushed; DADS is produced during the decomposition of allicin. DADS can easily be oxidized to allicin in the presence of peracetic acid or hydrogen peroxide. Similarly, allicin hydrolyses to produce diallyl trisulfide and DADS. When DADS reacts with liquid sulfur, it produces diallyl polysulfide mixtures that can create unbroken sulfur chains up to 22 atoms in length (Yi and Su, 2013).

An increasing number of studies have found that DADS exhibits anticancer activity against several kinds of tumor cells, including gastric cancer, breast cancer, and colon cancer cell lines (Altonsy et al., 2012; Tang et al., 2013; Xiao et al., 2014). Specifically, the underlying mechanism behind the anticancer action of DADS involves inducing apoptosis, activating metabolizing enzymes that detoxify carcinogens, inhibiting the production of reactive oxygen species, suppressing DNA adduct formation, and regulating cell cycle arrest. DADS can also suppress the metastatic potential of tumors such as EMT, invasion, and migration (Tsubura et al., 2012).

The purpose of this review is to evaluate the existing information on the potential of the anticancer activities exhibited by DADS as well as its mechanism of action, while also summarizing toxicity and pharmacokinetic studies conducted on this substance.

## 2 *In Vitro* and *In Vivo* Preclinical Studies

### 2.1 Breast Cancer

DADS has been successfully shown to intrinsically induce the apoptosis pathway in breast cancer (Fulda and Debatin, 2006; Lei et al., 2008; Lee et al., 2011b; Williams et al., 2018). The apoptosis pathway is regulated by several complex molecules that affect the production of distinct antiapoptotic and proapoptotic proteins. The upregulation of activating caspase occurs during mitochondria-mediated apoptosis, which is regulated by the proteins of the Bcl-2 family. Proapoptotic molecules (Bax, Bim, and Bad) co-exist with a variety of antiapoptotic proteins in the Bcl-2 protein family but can improve the expression of antiapoptotic proteins. Bcl-2 proteins prevent the permeabilization of the outer mitochondrial membrane and suppress apoptosis (Elmore, 2007; Tait and Green, 2010; Elumalai et al., 2012; Marie Hardwick and Soane, 2013; Redza-Dutordoir and Averill-Bates, 2016). In comparison to DADS, DADS-SLN resulted in the increased expression of the proapoptotic proteins Bad, Bax, caspase-3, and caspase-9, while also reducing the expression of antiapoptotic proteins, including Bcl-2. This shows that DADS-SLN causes apoptosis *via* intrinsic signaling (Talluri et al., 2017).

The percentages of late and early apoptosis in the control samples were 0.8% and 0.7%, respectively; this was considerably lower than the 55.6% and 3.3% exhibited by DADS-SLN. These values were also considerably larger than that of the blank SLN (0.9% and 1.4%) and DADS (12.42% and 2.61%), indicating that DADS-SLN enhanced apoptosis after medicating for 24 h at a concentration of 8 µM. The greater proportion of apoptotic cells in DADS-SLN compared to DADS suggests that the former has a much larger impact on apoptosis. Blank-SLNs appeared to have very little effect on the MCF-7 cells in terms of apoptosis; this minor cytotoxicity may be related to excipients. Thus, the nano-encapsulation of DADS might improve its anticancer impact, which is mostly associated with the increased induction of apoptosis (Talluri et al., 2017). In another trial, the late and early apoptosis cells in a control sample accounted for 0.9% and 0.6% of the cells, respectively. The study showed that DADS-RAGE-SLN improved the apoptotic activity of DADS in MDA-MB231 cells (Siddhartha et al., 2018).

Altonsy et al. (2012) found that DADS causes apoptosis in MCF-7 breast cancer cell lines by interfering with cell-cycle development stages such that the sub-G_0_ population increases and the synthesis of DNA are slowed significantly. In addition, DADS activates caspase-3 by inducing phosphatidylserine translocation from the inner to the outer leaflet of the plasma membrane. Additional research found that DADS regulates Bcl-w, Bcl-xL, Bcl-2, and Bax levels in cells in a dose-dependent manner, suggesting that the Bcl-2 proteins are involved in the apoptosis induced by DADS. Histone deacetylation inhibitors (HDACi) have been shown to reduce cancer cell growth and cause apoptosis. In the context of MCF-7 cells, DADS possesses HDACi characteristics because it prevents the loss of an acetyl group from the acetylated substrate and causes histone-4 (H4) hyperacetylation. Thus, the HDACi characteristics of DADS may be responsible for the activation of apoptosis in breast cancer cells (Altonsy et al., 2012). Another experiment found that miR-34a expression was upregulated in MDA-MB-231 cells that were treated with DADS. miR-34a suppressed breast cancer development both *in vitro* and *in vivo* while also enhancing the antitumor effectiveness of DADS. Specifically, miR-34a inhibits the expression of SRC, which results in the suppression of the Ras/SRC/ERK pathway. miR-34a can also be considered to be an effective agent for gene therapy procedures as it boosts the antitumor activity of DADS (Xiao et al., 2014).

DADS was used to pretreat (PreTx) MCF-10A cells in the presence of the carcinogen benzo(a)pyrene (BaP). MCF-10A cells were also co-treated (CoTx) with Bap and DADS for up to 24 h to evaluate the inhibitory influence of DADS on early carcinogenic events. The cells were monitored for any changes in the cell cycle, DNA damage, cell proliferation and viability, and the induction of peroxide formation. BaP tended to significantly increase cell proliferation at 6 h; DADS CoTx suppressed this phenomenon. Within 24 h, DADS prevented BaP-induced extracellular aqueous peroxide production; this behavior was independent of concentration or technique. Throughout DADS CoTx and PreTx, with notable suppression for every treatment sustaining after 6 h, DADS suppressed the single-strand break in DNA induced by BaP at all times. In normal cell lines, DADS was effective at inhibiting BaP-induced cell cycle transitions, cell proliferation, DNA damage, and the production of reactive oxygen species; thus, DADS may also suppress the environmentally-induced initiation of breast cancer (Nkrumah-Elie et al., 2012). The role of DADS, as well as the mechanisms behind its influence on breast cancer stem cells (BCSCs), were explored in a separate study. The findings showed that DADS reduced glucose metabolism and cell stemless in BCSCs. DADS also reduced the metastasis and the growth of BCSCs *in vivo* investigations by targeting the pyruvate kinase M2 (PKM2), CD44, and AMPK signaling pathways in BCSCs. An IHC analysis found that the expression levels of AMPK, CD44, and PKM2 were positively correlated in the tissues of 125 breast cancer patients. Furthermore, the positive expression of PKM2, AMPK, and CD44 was linked to poor overall survival and disease-free survival in patients (Xie et al., 2018). Furthermore, DADS caused the downregulation of MMP-9, the dysregulation of Bcl-2 family members, and the reversal of the epithelial-mesenchymal transition (EMT). Remarkably, DADS also suppressed the activation of the β-catenin signaling pathway, which is associated with the regulation of the Bcl-2 family, EMT, and MMP-9 in TNBC cells. The effectiveness of the anticancer properties of DADS was confirmed in MDA-MB-231 xenograft mice, which was consistent with the *in vitro* findings. Treating these mice with DADS appreciably lowered tumor weight and volume while raising apoptosis; in addition, active β-catenin expression levels were reduced and the downstream molecules underwent dysregulation (Table 1) (Huang et al., 2015).

**TABLE 1:** Possible antineoplastic properties of DADS as well as their underlying mechanisms as established by *in vitro* research.

Cell lines	Duration and conc.	Anticancer properties	Mechanism of action	References
Breast cancer				
MCF-7	8 µM (24 h)	Induced apoptosis	↑caspase-3, ↑Bax, ↑caspase-9, ↑Bad, ↓Bcl-2	Talluri et al. (2017)
MDA-MB231	5 µM (24 h)	Increased apoptosis	↑caspase-9, ↓Bcl2 and surviving, ↑ROS	Siddhartha et al. (2018)
Breast cancer stem cells (BCSCs)		Inhibited cell proliferation	↓glucose metabolism, ↓metastasis, ↓CD44, ↓PKM2, ↓AMPK	Xie et al. (2018)
MDA-MB-468, MDA-MB-231, and BT-549	50–400 μM (48 h)	Induced apoptosis	↓Bcl-2, ↓β-catenin signaling pathway, ↓MMP-9	Huang et al. (2015)
MCF7	0–100 µM	Induced apoptosis	↑sub-G0 population, ↓DNA synthesis, ↑phosphatidylserine translocation, ↑caspase-3, ↑Bax, ↑Bcl-xL, ↑Bcl-2, ↑Bcl-w, ↓Histone deacetylation (HDACi), ↑histone-4 (H4) Hyper-acetylation	Altonsy et al. (2012)
MDA-MB-231, MCF-10A	0–400 µM (48 h)	Inhibited proliferation of cells	↑miR-34a, ↓SRC/Ras/ERK pathway, ↓SRC	Xiao et al. (2014)
MDA-MB-231	50–1,200 µM (24 h)	Inhibited cell growth	↓TNF-α-induced release of CCL2 from triple-negative human breast tumor (MDA-MB-231) cells	Bauer et al. (2014)
MDA-MB-468	46.85–1,500.0 µM (24, 48 h)	Inhibited cell growth	↑caspase-3, ↑apoptosis, ↑NQO1, ↑SOD, ↑GSH	Sujatha et al. (2017)
MCF-7	100–400 µM (24 h)	Inhibited invasion and metastasis of cells	↓Vimentin, ↓MMP-9, ↑E-cadherin, ↓p38	Chen et al. (2016)
MDA-MB-231	100 µM (24 h)	Inhibited cell growth	↑NF-κB mRNA, ↓p38, ↓MEK, ↓TNF-α invoked CCL2 production, ↓IKKɛ, ↓MAPK/ERK, ↓NF-κB pathway signaling	Bauer et al. (2015)
MDA-MB-231	40 µM (24 h)	Inhibited cell proliferation	Not reported	Wei et al. (2017)
CMT-13	Less or equal 1,000 µM (72 h)	Suppressed cell growth	↓cell cycle G2/M phase	Tsubura et al. (2012)
MCF-7	7.62 mg	Induced apoptosis	↓ mitochondrial membrane potential, ↑ mitochondrial depolarization, ↑Caspase-3,↑ procaspase-3	An et al. (2015)
MCF-10A	6, 60, and 600 µM 24 h	Induced apoptosis	↓BaP-induced G2/M arrest, ↑extracellular aqueous peroxide, ↓BaP-induced DNA single-strand breaks, ↓reactive oxygen species	Nkrumah-Elie et al. (2012)
Esophageal cancer				
ECA109,L02	20, 40, 60 µg/ml (24 h)	Induced G2/M arrest and promoted apoptosis	↓cyclin B1, ↓cdc2, ↓p-cdc2, ↓cdc25c, ↑p53, ↑ p21	Yin et al. (2014b)
BAR-T	0–30 μg/ml (24, 48, and 72 h)	Abolished apoptotic resistance	↓NF-κB, ↓ROS, ↓IκBα phosphorylation, ↓ p50, ↓Bcl-2	Feng et al. (2017)
ECA109	0, 20 and 40 μg/ml (24 h)	Induced apoptosis	↓PCNA, ↓RAF/MEK/ERK, ↑caspase-3, ↑ p53, ↑Bax/Bcl-2 ratio	Yin et al. (2014a)
CE81T/VGH	50 μM (24 h)	Caused DNA damage	↓NAT1 mRNA, ↓protein levels of NAT	Yu et al. (2005)
Gastric cancer				
MGC803	30 mg/L (12, 24, 36 and 48 h)	Cell cycle arrest and apoptosis	↑phospho-Chk1 protein, ↑phospho-ATR expression	Ling et al. (2014)
AGS	0, 50, 100, 200, and 400 μM (48 h)	Induced apoptosis	↓percentage of live AGS cells and sub-diploid DNA content, ↓Bcl-2, ↑ Annexin V positive/PI negative area, ↑ROS production, ↑expressions of Fas, caspase-3, ↑ Bax	Lee et al. (2011a)
OE19	≤10 µg/ml (24-h)	Inhibited metastasis	↓MMPs, ↓NF-κB, and PI3K signaling pathways, ↑u-PA, ↑TIMPs	Yin et al. (2018)
AGS	100 mM (6 h)	Inhibited tumor cell motility and invasion	↓ MMP-2 and -9 mRNA and proteins, ↑ TIMP-1 and -2 mRNA levels and proteins	Park et al. (2011)
BGC-823, MGC-803, MKN-28, HGC-27, SGC-7901, and AGS	100 µM (48 h)	Suppressed proliferation and induced apoptosis	↑miR-200b, ↑miR-22	Tang et al. (2013)
MGC803	30 mg/L (12, 24, and 48 h)	Inhibited cell migration and invasion	↓p-LIMK1, ↓ p-cofilin1, ↓Rac1-Pak1/Rock1-LIMK1 pathway, ↓EMT, ↓MMP-9, ↑ TIMP-3	Bo et al. (2016)
MGC-803	20.30 and 40 mg/ml (24 h)	Halted cell migration and invasion	↑RORα, ↑nM23, ↑TIMP-3, ↑E-cadherin, ↓LIMK1, ↓uPAR, ↓CDK1 receptor, ↓ERK/Fra-1 pathway, ↓ MMP-9, ↓vimentin	Su et al. (2015)
BGC823	15 mg/L (12, 24, 36, and 48 h)	Significantly reduced the proliferation of cells	↓Cdc25C, ↓cyclin B1, ↑Chk1 phosphorylation, ↑phospho-ATR, ↑p21, ↑GADD45a, ↑p53	Ling et al. (2010), Bo et al. (2014)
MGC803	30 mg/L (0.12, 24 and 48 h)	exerted anti-EMT and antitumor growth effects	↓TGF-β1, ↓Rac1, ↓β-catenin, ↓vimentin, ↑E-cadherin	Su et al. (2018)
MGC803	30 mg/L (0, 6, 12, or 24 h)	Arrested cell cycle and inhibited cell proliferation	↑Acetylated histones H3 and H4, ↑p21^WAF1^ protein expression	Su, (2012)
Colon cancer				
SW480	0–500 µM (1 h)	Induced apoptosis and cytotoxicity	↑[Ca^2+^] concentration, ↑phospholipase C-independent Ca^2+^ release from ER, ↑extracellular Ca^2+^ influx	Chen et al. (2012)
HT-29	0, 30, 60, 120 and 240 μmol/L (12, 24 and 48 h)	Induced anti-proliferative and cytotoxic activity	↑expression of p21, ↑MM1, ↓ YWHae, ↓ RRM1	Lu et al. (2007)
HCT-116	50, 100, 200, and 400 μM (12.24 and 48 h)	Cell cycle arrest in the G2/M phase	↓ROS, ↓proliferation, ↑p53 expression, ↑cyclin B1	(Jo et al., 2008; Song et al., 2009)
Colo 205	25 μM (24-h and 48-h)	Elevated chemo-resistance	↑expression of drug resistant genes, ↑MRP3 gene expression	Lai et al. (2012)
Colo 205	10 and 25 μM	Inhibited migration and invasion	↓Ras, ↓PI3K, ↓p38, ↓MEKK3, ↓ERK1/2, ↓MKK7, ↓ JNK1/2, ↓MMP-2, -7, and -9	Lai et al. (2013)
SW480	85ppm (24 h)	Attenuated proliferation and induced apoptosis	↓GSK-3β; ↓NF-κB	Saud et al. (2016)
HT29	ED_50_ 69 µm (16 h)	Induced redox potrntial oxidation and reduced cell proliferation	↓cell proliferation, ↓reduced GSH, ↑oxidized GSH	Odom et al. (2009)
COLO 205	25 µM (24 h)	Induced apoptosis	↓the mitochondrial membrane potential, ↓Bcl-2, ↓ Bcl-xL, ↑Bak, ↑Bax, ↑cyclin B; ↑cdc25c-ser-216-9; ↑Wee1; ↑caspase-3, -8 and -9 activity, ↑Fas, ↑phospho-Ask1, ↑JNK, ↑p53, ↑cytochrome c	Yang et al. (2009)
HT-29	120 µM (12, 24 and 48 h)	Suppressed cell growth	↑p21, ↑MM1, ↓YWHAE, ↓RRM1	Huang et al. (2011)
CT26	100 μg/ml (1, 2, 4, and 8 h)	Enhanced cytotoxicity	↑dual functioning ability, ↑drug release, ↑ease of penetration through mucous membranes, ↑ cell cycle arrest at sub-G1 phase	Saraf et al. (2021)
SW620, SW480, and HCT116	1.008 g/ml (24 h)	Inhibited migration and invasion	↓Rac1	Xia et al. (2019)
HCT116, DLD-1, HT29, and SW620	100 µl (24 h)	Initiated apoptosis	↓Bcl-2, ↑Bak, ↑Bax, ↑caspase-9	Kim et al. (2019)
SW480	45 mg/L (24 h)	Inhibited the migration and invasion	↓phosphorylation of ADF/cofilin, ↓LIMK1, ↓vimentin; ↓CD34; ↓ Ki-67	Su et al. (2017)
Caco-2,HT-29	200 µM (6 h)	Increased histone acetylation and provided protective properties	↑p21^waf1/cip1^ expression, ↑histone H3 acetylation, ↑histone H4 hyperacetylation, ↓HDAC activity, ↓AM at the same	Druesne et al. (2004)
Caco-2, HT-29	200 µM (3 h and 6 h)	Increased histone acetylation and cell cycle arrest	↑CDKN1A promoter-associated histone acetylation, ↑p21^cip1^ mRNA and protein levels	Druesne-Pecollo et al. (2008)
Cervical cancer				
HeLa	10 µM 24 h	Inhibition of cell viability	↑apoptosis, ↓p73, ↓radiation-induced G2/M phase arrest, ↑Tap73, ↑ΔNp73, ↑FASLG, ↑APAF1	Di et al. (2015)
HeLa	0–100 µM 24 h	Cell growth inhibition	↑sub-G1 phase (apoptosis), ↑ G0/G1 cell cycle arrest, ↑dysfunction of mitochondria, ↑DNA damage, ↑cytochrome c,↑pro-caspase-3 and -9, ↑AIF, ↑Endo G	Wu et al. (2011)
Caski	-	Inhibited cell proliferation	↑Intracellular ROS, ↑apoptosis,↑cell cycle arrest in G0/G1 phase, ↓cyclin D1, ↓CDK4, ↑p21WAF1/CIP1, ↑p27KIP1, ↓E6, ↓E7	Ansari et al. (2020)
Ovarian cancer				
SK-OV-3 and OVCAR-3 ovarian	30 mg/L	Inhibited cell proliferation	↑p-Chk1, ↑p-CDC25C, ↑p-P53, ↑P21WAF1, ↑p-CDK1, ↓CDK1, ↓CyclinB1 protein, ↓PCNA, ↓Ki-67, ↓Survivin, ↑Cleaved-caspase3	Zhang et al. (2019b)
Leukemia				
K562 and NB4	0, 25, 50, 75, 100, 200, 300, and 500 μg/ml (24 and 48 h)	Induced apoptosis and autophagy	↓cell viability, ↓mTOR expression, ↑the percentage of cell apoptosis	Suangtamai and Tanyong, (2016)
HL-60	4, 8, 16, 32, 64 and 128 µM (72 h)	Inhibited proliferation, migration, invasion and arrested cells at G0/G1 stage	↑differentiation, ↑CD11b expression, ↓NBT, ↓DJ-1; ↓cofilin 1; ↓RhoGDI2; ↓calreticulin (CTR) and PCNA	Ling et al. (2017)
HL-60	500 µl (24, 48 and 72 h)	Inhibited migration and invasion	↓DJ-1, ↓p-Src, ↓p-Fak	Liu et al. (2018)
HL-60	8 µM (72 h)	Suppressed proliferation of cell	↑CD11b and CD33 expression, ↓ cofilin 1, ↓phosphorylated cofilin 1, ↓Rac1, ↓ ROCK1, ↓ LIMK1, ↓ phosphorylation of LIMK1	Ling et al. (2020)
HL-60	5, 10, 15 mg ⁄ L (24 h)	Induced apoptosis	↑Rac2 gene, ↑NADPH oxidase, ↑ROS, ↑JNK	Yi et al. (2010)
HL-60	1.25 mg/L (8 h)	Induced apoptosis	↓cytoplasmic DJ-1 protein expression	Li et al. (2016)
	1.25 mg/L (8 h)		↑nuclear DJ-1 protein expression	
	5 and 10 mg/L (4, 8 or 12 h)		↓mitochondrial DJ-1 protein expression	
HL-60	1.25 mg/L (48 h)	Decreased proliferation,differentiation and invasion	↓CRT	Yi et al. (2016)
HL-60	1.25 mg/L (48 h)	Reduced cell proliferation, invasion, and differentiation	↓CRT, ↓ CD33, ↑C/EBPα, ↑ROS	Sun et al. (2019)
HL-60	20 micro mol/L (12 h)	induced the G(2)/M arrest	↑phospho-p38 MAPK, ↑phospho-Cdc25B, ↑phospho-Cdc2	Tan et al. (2004)
HL-60	1.25 μg/ml (24 h)	Triggered apoptosis	↑ROS, ↑PKCδ cleavage	Agassi et al. (2020)
HL-60	0.625, 1.250, and 2.500 μg/ml (24, 48, and 72 h)	Inhibited the proliferation	↓ VEGF mRNA, ↑VEGF protein	Fan et al. (2006)
KF62	10, 20, 40, 80 mg/L (48 h)	Induced autophagy	↑p-ERK, ↑LC3-Ⅱ	Ye and Yin, (2017)
Lymphoma				
U937	50 μM (24 h)	Induced apoptosis	↓hTERT, ↓DNA-binding activity of c-Myc and Sp-1, ↑Mad1, ↑Mad/Max complex	Dasgupta and Sengupta Bandyopadhyay, (2015)
Lung cancer				
A549	0–80 μM (24 h)	inhibited cell proliferation	↓MMP-2/9, ↑E-cadherin, ↓N-cadherin, ↓Nrf2 signaling	Xu et al. (2021)
A549	7.5 μM and 10 μM 24 h	Decreased cell viability	↓gelatinases, ↑E-cadherin, ↑cytokeratin-18, ↓N-cadherin and ↓vimentin	Das and Sinha, (2019)
Neural cancer				
SH-SY5Y, HeLa	50 µM (3 h)	Caused early morphological changes	↑SOD1, ↑ROS, ↑PP1-mediated Tau dephosphorylation	Aquilano et al. (2010)
SH-SY5Y	100 µM (24 h)	Induced apoptosis	↑[Ca^2+^], ↑cytosolic Smac/Diablo, ↑caspase-9, ↑caspase3, ↑Calpain, ↑SBDP, ↓NF-κB, ↓ICAD	Karmakar et al. (2007)
SH-SY5Y	50 µmol/L (24 h)	Caused nuclear damage, protein oxidation, and lipid peroxidation	↑ROS, ↑NO	Aquilano et al. (2007)
CCF STTG1, SW1783, SW1088, CHLA-03-AA	15 and 150 μg/ml (24 h)	Triggered apoptosis	↓Akt/PKB	Choromanska et al. (2020)
T98G and U87MG	100 μM (24 h)	Induced apoptosis	↑ROS, ↑phosphorylation of p38 MAPK, ↑JNK1 pathway, ↑[Ca^2+^], ↑calreticulin, ↑caspase-4, ↑caspase-9, ↑caspase-3, ↑Bax, ↑cytochrome, ↑Smac, ↑calpain, ↓Bcl-2, ↓BIRC proteins	Das et al. (2007)
Prostate cancer				
PC-3	10–50 μM	Inhibited cell growth	↑apoptosis, ↑IGF, ↓ phosphorylation of Akt, ↑cyclin D1, ↑Bcl-2 molecule ↑Bad, ↑NF-kB, ↑ Bax	Arunkumar et al. (2012)
LNCaP	200, 400 µM 24 h	Inhibited cell growth	↑TER, ↓claudin, ↓(MMP)-2 and -9	Shin et al. (2010)
PC-3	50–1,000 µM	Increased cytotoxicity	↓thapsigargin, ↑ apoptosis, ↑ROS, ↑[Ca2+], ROS production	Chen et al. (2011)
DU145	200, 400 µM 24 h	Inhibited cell growth	↑Apoptosis, modulation of ↑Bcl-2, ↓IAP, ↑DR4, ↑FasL, ↓Bid proteins, ↑phosphorylation of MAPKs	Shin et al. (2012)
PC-3	20, 40 µM 24 h	Decreased cell viability	↓NF-κB, ↓IKKα, ↓IKKβ	Arunakaran et al. (2013)

Bauer et al. (2014) demonstrated the anticancer properties by showing a prominent expression profile for the sustained release of IL-8, IL-6, plasminogen activator inhibitor-1, and TIMP1/2 in untreated/resting MDA-MB-231 cells using an initial chemokine/adipokine protein panel microarray. TNF-α (40 ng/ml) did not affect most of these molecules, except for a single large increase in CCL2 release (an approximately 1,300-fold upregulation). A sub-lethal dose of DADS (100 μM) inhibited and reversed the release of TNF-α-induced CCL2 (Bauer et al., 2014).

### 2.2 Gastrointestinal Tract and Associated Cancers

#### 2.2.1 Esophageal Cancer

EC is the sixth most frequently occurring cancer. It has a terrible prognosis worldwide and affects about 450,000 people. At one point, squamous cell carcinoma accounted for around 90% of EC cases, although epidemiology has seen a shift in various western nations (Epidemiologic differences in esophageal cancer between Asian and Western populations; Kamangar et al., 2009; Sadjadi et al., 2010; Yang et al., 2020). The occurrence of esophageal adenocarcinoma (EAC) has been gradually increasing over the last 30 years, and currently outnumbers squamous cell cases (Dabrowski et al., 1998; Comprehensive and Network, 2014). One study showed that DADS halted cancerous cells in the G2/M phase by modulating proteins that were related to the cell cycle; this modulation was linked to a decrease in the production of cyclin B1, cdc25c, cdc2, and p-cdc2. Furthermore, DADS regulated cellular apoptosis by upregulating the ratio of Bax to Bcl-2, activating caspase-3, and downregulating the MEK-ERK signaling pathway. The activation of the p53/p21 pathway involves the suppression of the G2/M phase, the induction of apoptosis, and the inhibition of cell differentiation. Hence, DADS controlled esophageal squamous cell carcinoma (ESCC) cells *via* many signaling pathways and was revealed to be a prospective anticancer therapy for ESCC (Yin et al., 2014b).

Feng et al. (2017) investigated the chemopreventive effectiveness of DADS against Barrett’s esophagus (BE) as well as any potentially linked signaling pathways by treating BAR-T cells with deoxycholic acid (DCA) in the absence or presence of DADS. DADS was not observed to decrease cell viability for a given range of concentrations. Like PDTC, an NF-κB inhibitor, DADS suppressed the ROS production induced by DCA, IκBα phosphorylation, inflammation, and the production of p50 in the nucleus in a dose-dependent manner. By lowering the amount of Bcl-2, DADS also boosted the rate of cell apoptosis. DADS had a minimal cytotoxic effect on BAR-T cells. DADS exhibited an anti-inflammatory effect against BAR-T cells through the NF-κB signaling pathway and ROS inhibition. Furthermore, DADS inhibited the DCA-induced resistance of apoptosis *via* a mechanism that is NF-κB/Bcl-2 dependent, suggesting that it could be a promising option for the chemical prevention and treatment of EAC and BE (Feng et al., 2017). Yin et al. (2014a) found that DADS dramatically decreased the viability of human esophageal cancer ECA109A cells while being considerably less toxic to normal liver cells. Annexin V-FITC/propidium iodide (PI) staining identified the proapoptotic impact of DADS on ECA109 cells. Flow cytometry analysis revealed that DADS enhanced apoptosis in a dose-dependent manner and that the caspase-3 inhibitor Ac-DEVD-CHO could reduce the rate of apoptosis. In a xenograft trial conducted on nude mice, DADS therapy decreased the development of ECA109 tumors at concentrations of 20 mg/kg and 40 mg/kg with no apparent adverse effects. By suppressing the proliferation of nuclear antigen (PCNA) cells, DADS reduced the proliferation of ECA109. In ECA109 xenograft tumors, DADS activated a mitochondria-dependent network with the executor of caspase-3, increased the ratio of Bax and Bcl-2, increased p53 levels, and downregulated the ERK/MEK/RAF pathway. These findings suggest that DADS is an efficient and reliable anti-cancer agent against esophageal carcinoma (Table 1) (Yin et al., 2014a).

#### 2.2.2 Gastric Cancer

With over an estimated one million new cases diagnosed in 2020 and 769,000 predicted deaths, gastric cancer remains a serious health concern across the world and ranks fifth in terms of its incidence and fourth in terms of its lethality (UICC, 2020; Sung et al., 2021). Its etiology might be influenced by several factors, including genetics and the environment. Garlic consumption is a commonly prescribed means of preventing gastric cancer (Wang et al., 2012; Shamshirian et al., 2018). Ling et al. (2014) showed the anticancer properties of DADS that it induced the buildup of phosphorylated Chk1 and had a downregulating effect on the expression of cyclin B1 and CDC25C. The overexpression of Chk1 resulted in a significant increase in G2/M arrest induced by DADS, the inhibition of CDC25C expression, and an increase in DADS-mediated Chk1 phosphorylation. Chk1 suppression decreased DADS-associated G2/M arrest and prevented the DADS-induced suppression of cyclin B1 and CDC25C. In addition, Chk1 signaling *via* CDC25C/Chk1/ATR/cyclin B1 mediated the DADS-induced G2/M checkpoint responses (Ling et al., 2014). By triggering apoptosis and increasing ROS generation, DADS considerably reduced the growth of AGS human gastric adenocarcinoma cells. DADS also decreased the expression levels of Bcl-2 in AGS cells while also increasing the expression levels of Bax, Fas, and caspase-3 (Lee et al., 2011a). DADS hindered the metastasis of type II esophageal-gastric junction adenocarcinoma cells (OE19) by suppressing the Akt/PI3K and NF-κB signaling pathways, downregulating uPA, MMP-9, and MMP-2 in the process (Yin et al., 2018). DADS inhibited cell proliferation by increasing tissue inhibitors of metalloproteinase (TIMP)-1 and TIMP2 mRNA levels and proteins while decreasing claudin proteins (claudin-3, claudin-4, and claudin-2), which are important components of tight junctions (TJs) (Park et al., 2011).

Tang et al. (2013) found that DADS inhibits cell proliferation and causes apoptosis *via* the Wnt-1 signaling pathway by upregulating miR-22 and miR-200b. Variations in the expression of miRNA were identified in DADS-treated MGC-803 cells, while the upregulation of miR-22 and miR-200b was also observed. Wnt-1 was also discovered to be a target of both miR-22 and miR-200b. miR-200b and miR-22 not only synergistically decreased gastric cancer development but also improved the antitumor effects of DADS both *in vitro* and *in vivo*. Thus, it was suggested that miR-22 and miR-200b could be used in possible gene therapy procedures to boost the antitumor effectiveness of DADS (Tang et al., 2013). By downregulating LIMK1, DADS prevented the epithelial-mesenchymal transition (EMT), preventing gastric cancer invasion and development. The expression of LIMK1 is linked with the differentiation, clinical stage, invasion level, lymph node metastasis, and poor diagnosis of tumors, both *in vitro* and *in vivo* (Bo et al., 2016). To investigate the potential of DADS-regulated molecules, Su et al. (2015) compared the protein expression profiles of DADS-treated gastric cancer MGC-803 cells to untreated control cells. 23 proteins with statistically important variations in expression were found through proteomic approaches, comprised of 14 downregulated proteins and 9 upregulated proteins. Ling et al. (2010) demonstrated that there was an association between the cell cycle arrest of G2/M and the phosphorylated forms of Chk1 buildup, but not Chk2; this suggests that the G2/M cell cycle arrest was associated with the DADS-induced growth suppression of the BGC823 gastric cancer cell line in humans. Additionally, Chk1 signaling *via* the Cdc25C/Chk1/cycling B1/ATR pathway mediates the DADS-induced G2/M checkpoint response; this process is independent of Chk2 (Ling et al., 2010). Bo et al. (2014) reported similar results; they found that Chk1, and not Chk2, was directly responsible for the arrest of G2/M, which is induced in the human gastric cancer BGC823 cells by DADS (Table 1). An *in vitro* trial conducted by Su (2012) found that DADS increased the acetylation of the H4 and H3 histones in human gastric cancer MGC803 cells in a time-dependent manner; this was supported by an increase in p21WAF1 protein levels increase and was consistent with the arrest of the G2/M phase cell cycle. DADS also exhibited dose-dependent antitumor effectiveness in an *in vivo* trial of MGC803-xenografted nude mice, resulting in the inhibition of tumor cell development and the arrest of the G2/M phase. Furthermore, the xenografted tumor cells exhibited distinct cell differentiation characteristics. These findings suggest that DADS, by causing the hyperacetylation of histones H4 and H3 while also boosting the expression of p21WAF1 in MGC803 cells, might trigger the arrest of the cell cycle and reduce cell proliferation, which may be responsible for its antitumor effects against gastric cancer (Su, 2012).

#### 2.2.3 Colorectal Cancer

Well over 1.9 million new cases of colorectal cancer (CRC) are expected to be diagnosed in 2020, with an estimated 935,000 fatalities. CRC is the third most frequently-occurring cancer and has the second-highest mortality rate (1. Hyuna Sung et al., 2020; UICC, 2020). A lack of exercise or physical activity, obesity, as well as the consumption of alcohol, red meats, and tobacco are all known to contribute to the development of CRC. CRC fatalities can be reduced if it is detected and treated early. The use of garlic as a dietary supplement can help prevent and lower the risk of CRC (Fleischauer and Arab, 2001). DADS had a preliminary signaling impact on the SW480 colon cancer cell line by increasing Ca^2+^ mobilization (Chen et al., 2012). The DADS-induced apoptosis of colo205 cells was related to the elevated expression of transcription 1 (STAT1) signal activators and transducers (Lu et al., 2007). In HCT-116 cells, DADS triggered the arrest of the cell cycle in the G2/M phase, while also elevating the expression of p53 and cyclin B1 (Jo et al., 2008), as well as the production of ROS (Song et al., 2009). DADS targeted drug-resistant genes in human colon cancer cells (colo205). The expression levels of the multidrug resistance-associated protein-3 (MRP-3) were increased by DADS. DADS elevated the expression of the MRP6 and MRP4 genes while also inhibiting the growth of colo205 cells by lowering the Ras, PI3K, MEKK3, MKK7, JNK1/2, p38, and ERK1/2 expression levels, which consequently suppressed MMP-9, MMP-2, NF-κB, COX-2, and MMP-7 expressions levels (Lai et al., 2012, Lai et al., 2013).

DADS prevented colorectal tumorigenesis in mouse models through a process that involved the NF-κB signaling pathway and the nuclear localization of NF-κB, causing it to have a reduced activity while also inhibiting the activation of GSK-3β (Saud et al., 2016). An *in vivo* study using human colon cancer colo205 cells transplanted into mice revealed that DADS decreased the tumor’s weight and size (Lai et al., 2012). DADS dramatically decreased cell proliferation and triggered the arrest of the cell cycle in the G2/M phase in human colon cancer SW480 cells both *in vitro* and *in vivo*; this was most likely accomplished by downregulating cyclin B1, p53, and PCNA expression while upregulating p21WAF1 (Liao et al., 2007). Through the alteration of its intracellular redox environment, DADS suppressed the growth and arrest of the G2/M phase in human colon cancer HT-29 cells (Odom et al., 2009; Yang et al., 2009). The anti-proliferative properties of DADS against colon cancer HT-29 cells were linked to several genes that had varied expression patterns and that were engaged in different physiological systems (Huang et al., 2011). Colon-targeting DADS-loaded nanoparticles that had dual functionalities and a significant cytotoxic impact were successfully produced. In colon cancer therapy, ES100/PLGA-NPs might be a viable method of targeting water-insoluble bioactive phytochemicals with better safety measures and patient compliance. Furthermore, the polymeric mixture used can be precisely adjusted to create nano-carriers that can deliver dietary phytochemicals (Saraf et al., 2021).

DADS inhibited the expression levels and activity of Rac1 by inhibiting the PI3K/Akt pathway, preventing EMT as well as cell migration and invasion. The Rac1 knockdown improved the tumor prevention capabilities of DADS; in contrast, the overexpression of Rac1 counteracted its effects (Table 1) (Xia et al., 2019). In CRC cell lines, Kim et al. (2019) found that non-cytotoxic doses of DADS boosted tumor necrosis factor-related apoptosis-inducing ligand (TRAIL)-related cell death. In addition, the synergistic impact between TRAIL and DADS was confirmed *in vivo* using mice models. One of the mechanisms involved in these observations was the reduced production of the antiapoptotic protein Bcl-2; the synergistic effect of DADS and TRAIL was diminished in cells with overexpressed Bcl-2. This study revealed new insights into the involvement of DADS in the TRAIL-associated suppression of CRC development *via* Bcl-2 inhibition (Kim et al., 2019). The overexpression of LIMK1 considerably aids colon cancer cell invasion and migration. DADS reduced cancer cell invasion and migration by decreasing the phosphorylation of ADF/cofilin *via* the suppression of LIMK1 in colon cancer cells. The knockdown and overexpression of LIMK1 increased and decreased DADS-induced cell proliferation suppression, respectively; this was confirmed both *in vitro* and *in vivo*. Similar results were observed in DADS-induced alterations to the expression of E-cadherin, Ki-67, CD34, and vimentin in xenografted tumors. These findings suggested that LIMK1 was a viable target molecule to enhance the anti-invasion and anti-migration effects of DADS on colon cancer cells (Su et al., 2017). By inhibiting histone hyperacetylation and HDAC while increasing the expression of cip1/p21waf1, DADS suppressed cell proliferation in HT-29 and Caco-2 cells. The cellular and molecular effects evoked by DADS are potentially connected to its impact on histone acetylation and thus contribute to its anti-carcinogenic capabilities in the colon (Druesne et al., 2004). In both short-term, solitary treatments as well as persistent, repetitive treatments, DADS caused rapid histone hyperacetylation in human tumor colon cell lines. Histone hyperacetylation is linked to anti-proliferative effects, including the arrest of the cell cycle in the G2/M phase and the increase of p21cip1 mRNA and protein expression levels (Druesne-Pecollo et al., 2008).

#### 2.2.4 Hepatocellular Cancer

There were an estimated number of 906,000 new cases and 830,000 fatalities attributed to primary liver cancer in 2020. It is the most common form of cancer, has the highest mortality rate, and is the sixth most frequently diagnosed cancer worldwide (Anwanwan et al., 2020; Globocan, 2020; Sung et al., 2021). The two treatments available for liver cancer are immunotherapy and chemotherapy. However, new therapeutic approaches that incorporate natural ingredients may result in better prognoses. In particular, garlic has exhibited anticancer and preventative effects against liver cancer (Zhou et al., 2016). Through inhibition of IĸBα phosphorylation and NF-ĸB translocation in CCL4-induced liver injury, DADS boosted the levels of phase II antioxidant enzymes while simultaneously decreasing the expression of inflammatory mediators. This suggests that DADS activated the Nrf2 pathway, which enhanced antioxidant defense, inhibited NF- ĸB activation, and decreased inflammation (Lee et al., 2014). Another study found that DADS successfully reduced the acute hepatic damage caused by acetaminophen in rats. The beneficial impacts of DADS are due to its ability to regulate inflammatory reactions by suppressing NF-κB activation and reducing oxidative stress-mediated JNK activation by inhibiting CYP2E1 or by increasing antioxidant enzyme activity (Table 1) (Ko et al., 2017). DADS suppressed the critical regulators of lipid peroxidation, inflammation, and metabolism, while also having appreciable effects on nonalcoholic steatohepatitis (NASH) induced by high-fat diets (HFD) or methionine and choline-deficient diets (MCD) (Zhang et al., 2019a). The administration of DADS reduced cyclophosphamide-induced hepatotoxicity in rats by simultaneously upregulating both anti-inflammatory and antioxidant activity, demonstrating its promising therapeutic utility against the adverse effects of cyclophosphamide (Hasan et al., 2020). The pre-treatment of epithelial cells in CdCl_2_-treated rat liver with DADS compounds exhibited a protective effect against the toxicity of CD by modulating cytokine protein production, which resulted in improved viability (Odewumi et al., 2019).

Table 1 demonstrates the possible antineoplastic properties of DADS as well as their underlying mechanisms as established by *in vitro* research.

### 2.3 Gynecological Cancers

#### 2.3.1 Cervical Cancer

Cervical cancer is the fourth most common cancer, with 342,000 fatalities and 604,000 new cases diagnosed in women globally in 2020. Middle and low-income nations accounted for over 90% of cases worldwide (Sung et al., 2021; Mitra et al., 2022b). Thus, therapeutic treatments that use natural products are urgently needed. Several experimental studies have shown that DADS is effective against cervical cancer. Di et al. (2015) investigated the molecular mechanisms associated with DADS using human cervical cancer cells. As radiotherapy is the most basic form of treatment, HeLa cells were treated with 10 µM of DADS before being exposed to radiation, increasing their radiosensitivity and reducing cell viability. DADS pre-treatment reduced the radiation-induced arrest of the G2/M phase while also boosting radiation-induced apoptosis. In addition, coupled DADS and radiation treatment boosted the activation of apoptosis pathways as well as increased the Tap73 (proapoptotic) to Np73 (antiapoptotic) ratio and the levels of APAF1 and FASLG downstream proteins (Di et al., 2015). DADS had a substantial anti-proliferative impact on Caski cells, dose-dependently decreasing cell viability and increasing intracellular ROS production and apoptosis. Downregulating cyclin CDK4 and D1 and the overexpression of p27KIP1 and p21WAF1/CIP1 inhibitors of CDK enhanced the ability of DADS to arrest the G0/G1 cell cycle in Caski cells. DADS also downregulates the viral oncogenes E7 and E6 while restoring the functions of p53 (Ansari et al., 2020).

#### 2.3.2 Ovarian Cancer

Ovarian cancer is the seventh most frequent disease in women across the globe and the 18th most prevalent cancer. In 2012, there were almost 239,000 new instances of ovarian cancer in women, which accounts for approximately 4% of all new cancer cases in women (2% of all cancer cases across both genders). Ovarian cancer is generally lethal. The age-standardized prevalence rate of this cancer ranges from less than 5 per 100,000 in Africa to more than 11 per 100,000 in Eastern and Central Europe (Sánchez et al., 2013). To mitigate the extreme prevalence of ovarian cancer, novel therapeutics must be considered. DADS has been used for decades to treat this disease. SK-OV-3 and OVCAR-3 cells were incubated with different concentrations of DADS to explore the influence of the DADS-induced arrest of the G2/M phase on the development and death of ovarian cancer cells as well as the molecular mechanisms that are involved in this process. The test was run on xenograft models *in vivo* which demonstrated that the inhibition rate of cell proliferation dramatically increased as the concentration of DADS increased. The suppression rates of the OVCAR-3 and SK-OV-3 cells were noticeably greater at 24 h than at 12 and 48 h, demonstrating remarkable time-dependency. Another test demonstrated that when OVCAR-3 and SK-OV-3 cells were medicated with DADS of various concentrations, the rates of apoptosis increased as the concentration of DADS. Notably, the apoptosis rates of DADS treated OVCAR-3 and SK-OV-3 cells at 24 h were much greater than the rates at 12 and 48 h. The intraperitoneal injection of a solution containing DADS significantly reduced the volume of xenografted cancer cells in the ovaries of nude mice compared to the blank control groups. When 30 mg/L of DADS was administered to OVCAR-3 and SK-OV-3 cells for 24 h, the proportion of OVCAR-3 and SK-OV-3 cells in the G2 phase rose dramatically compared to the blank cells. Survivin, PCNA, and Ki-67, which are associated with cell apoptosis and proliferation, were all dramatically reduced while the levels of the protein cleaved-caspase3 significantly increased [54].

### 2.4 Hematological Cancers

#### 2.4.1 Leukemia

The type of leukemia is determined by the type of malignant blood cell and the rate at which it proliferates (Van Den Heuvel-Eibrink, 2004). Leukemia is the most frequent cancer in patients older than 55 while also being the most prevalent illness in children younger than 15 years old (Deschler and Lübbert, 2008). In the cell lines of NB4 and K562 myeloid leukemia, autophagy and apoptosis could both be triggered by DADS *via* the mTOR pathway (Tan and Xiao-xia, 2011). After treatment with DADS at different concentrations for 24–48 h, it was found that DADS suppressed cell viability and increased the apoptosis rate in a time- and dose-dependent manner. The expression of mTOR was significantly reduced in cells that had been treated with DADS and mTOR inhibitors. Cells treated with 10 µM of mTOR inhibitor and 100 g/ml of DADS exhibited high rates of autophagy and death (Suangtamai and Tanyong, 2016). DADS halted cells at the G0/G1 stage and reduced the proliferation, invasion, and migration of HL-60 cells. DADS also lowered the capacity of NBT, improved the expression of CD11b, slowed tumorigenesis, and promoted differentiation in xenografts *in vivo* (Table 2). The expression of Rho GDP dissociation inhibitor 2 (Rho GDI2), Dj-1, Calreticulin (CTR), PCNA, and cofilin 1 were all lowered by DADS (Ling et al., 2017). DADS suppressed DJ 1-mediated migration and invasion in leukemic cells by inhibiting the Src-Fak-Integrin signaling pathway; the Src inhibitor synergistically improved the anticancer effects of DADS (Liu et al., 2018). Due to the reduced ability of nitro-blue tetrazolium as well as elevated CD33 and CD11b expressions, 8 µM of DADS inhibited cell migration, proliferation, and invasion, while also causing differentiation. DADS significantly decreased the generation of phosphorylated cofilin 1 in HL 60 leukemia cells. DADS also decreased the protein and mRNA expression of Rac1, LIM domain kinase 1 (LIMK1), and Rho-associated protein kinase 1 (ROCK1), as well as LIMK1 phosphorylation in HL 60 cells (Ling et al., 2020).

**TABLE 2:** Potential antineoplastic effects of DADS and its underlying mechanisms based on *in vivo* studies.

Animal tumor models	Anticancer effects	Mechanisms	Dose (route)	Duration	References
Breast cancer					
Female athymic mice (breast)	Retarded the tumor growth	Not reported	Intraperitoneal injection (1 or 2 mg) 3 times a week	35 days	Tsubura et al. (2012)
Ehrlich ascites carcinoma (EAC) bearing female albino mice	Modulated apoptosis	↑Apoptosis, ↓Bcl-2, ↑p53, ↑deoxynucleotidyl transferase, ↓sialic acid	Intraperitoneally 100 mg/kg	2 weeks	Ahmed and Ahmed, (2015)
Esophageal cancer					
ECA109 injected nude mice	Induced apoptosis	↓PCNA, ↓RAF/MEK/ERK, ↑caspase-3, ↑p53, ↑Bax/Bcl-2 ratio	20 and 40 mg/kg (i.p.)	24 h	Yin et al. (2014a)
Gastric cancer					
Male Balb/c nude mice	Induced apoptosis	↑miR-22, ↑miR-200b	100 mg kg^−1^ (s.c.)	48 h	Tang et al. (2013)
MGC803 injected nude mice	Inhibited cell invasion	↓ p-cofilin1, ↓Rac1-Pak1/Rock1-LIMK1 pathway, ↓EMT, ↓p-LIMK1, ↓MMP-9, ↑ TIMP-3	30 mg/L (s.c.)	12, 24, and 48 h	Bo et al. (2016)
MGC803 injected male athymic BALB/c nude mice	Exerted anti-EMT and antitumor growth effects	↓Ki-67, ↓CD34, ↓vimentin, ↑E-cadherin	30 mg/L (s.c.)	0.12, 24 and 48 h	Su et al. (2018)
MGC803 -xenografted nude mice	Arrested cell cycle and inhibited cell proliferation	↑Acetylated histones H3 and H4, ↑p21^WAF1^ protein expression	50, 100, and 200 mg/kg (s.c.)	0, 6, 12, or 24 h	Su, (2012)
Colon cancer					
Colo 205 xenograft mice	Elevated chemo-resistance of human cancer cells	↑Mdr1, MRP1, MRP3, MRP4 and MRP6 gene expression	25 μM (s.c.)	24-h and 48-h	Lai et al. (2012)
FVB/N mice	Prevented colorectal tumorigenesis	↓prolonged inflammation and cellular transformation; ↓GSK-3β, ↓NF-κB	10 mg/kg (i.p.)	24 h	Saud et al. (2016)
SW480 injected nude mice	Inhibited proliferation and arrested cell cycle	↓PCNA, ↓p53, ↓cyclin B1, ↑p21WAF1	30 mg/kg (s.c.)	24 h	Liao et al. (2007)
SW620, SW480, and HCT116 injected nude mice	Inhibited migration and invasion	↓Rac1, ↓N-cadherin, ↓vimentin, ↓snail1, ↑E-cadherin	1.008 g/ml (i.v.)	24 h	Xia et al. (2019)
HCT116, DLD-1, HT29, and SW620 injected BALB/c nude mice	Initiated apoptosis	↓Bcl-2, ↑Bak, ↑Bax, ↑caspase-9	100 µL (s.c.)	24 h	Kim et al. (2019)
SW480 injected nude mice	Inhibited the migration and invasion	↓phosphorylation of ADF/cofilin, ↓LIMK1, ↓vimentin, ↓CD34, ↓ Ki-67	45 mg/L (s.c.)	24 h	Su et al. (2017)
Caco-2,HT-29 injected rodents	Increased histone acetylation and provided protective properties	↑p21^waf1/cip1^ expression, ↑histone H3 acetylation, ↑histone H4 hyperacetylation, ↓HDAC activity, ↓AM at the same	200 µM (s.c.)	6 h	Druesne et al. (2004)
Caco-2, HT-29 injected colonocytes	Increased histone acetylation and cell cycle arrest	↑CDKN1A promoter-associated histone acetylation, ↑p21^cip1^ mRNA and protein levels	200 mg/kg (Intracaecal perfusion and gavage)	1 and 21 h	Druesne-Pecollo et al. (2008)
Hepatocellular cancer					
Thirty male Sprague-Dawley rats	Induced antioxidant defense mechanism and reduced inflammatory response	↓NF-ĸB translocation, ↓ IĸBα phosphorylation, ↑Bax, ↑cytochrome c, ↑caspase-3, ↑Nrf2 translocation, ↑phase II/antioxidant enzyme activities	50 and 100 mg/kg/day (gavaged)	5 days	Lee et al. (2014)
Twenty four healthy male rats	Provided protective effects	↓hepatic CYP2E1 expression, ↓NF-κB activation, ↓serum AST and ALT levels, ↓MDA, ↓JNK activation, ↑GSH, ↑antioxidant enzymes activities	100 mg/kg/day (oral gavage)	5 days	Ko et al. (2017)
c57Bl/6J mice	Effectively attenuated hepatic steatosis, lipotoxicity, lipid peroxidation and inflammation	↓serum AST and ALT levels, ↓liver TG and TC contents, ↓ mRNA levels of SREBP-1 and Apoa-I, ↓SCD-1, ↓ NF-κB, ↓ TNF-α, ↓IL-6, ↓MDA, ↓SOD, ↑PPARα, ↑mRNA levels of CREBH and FGF21	20, 50, and 100 mg/kg (s.c.)	4 or 20 weeks	Zhang et al. (2019a)
CP feeded male adult albino rats	Reduced hepatotoxicity	↑ALT, ↑AST, ↑ALP, ↑ total and direct bilirubin levels, ↑γ-GT, ↑HDL-C, ↑GPx, ↓serum cholesterol, ↓ triglycerides, ↑CAT, ↓LDL-C, ↓ VLDL-C levels, ↓MDA, ↓PCC, ↓NOX-4	200 mg/kg (oral)	10 days	Hasan et al. (2020)
CRL1439 treated rats	Triggered apoptosis	↓IGF-1R, ↓Fas/TNFRSF6/APO	150 µM (oral)	2 h	Odewumi et al. (2019)
Leukemia					
HL-60 injected mice	Inhibited proliferation, migration, invasion and arrested cells at G0/G1 stage	↑differentiation, ↑ CD11b expression, ↓NBT, ↓DJ-1, ↓ cofilin 1, ↓RhoGDI2, ↓CTR, ↓PCNA	21, 42 and 84 mg/kg (s.c.)	5 days	Ling et al. (2017)
HL-60 injected SCID mice	Reduced cell proliferation, invasion, and differentiation	↓CRT, ↓CD33, ↑C/EBPα, ↑ROS	21 or 42 mg/kg (s.c.)	21 days	Sun et al. (2019)
Skin cancer					
DMBA/TPA-treated mouse	Inhibited chemically induced papilloma genesis	↑CAT, ↑SOD, ↑GPx, ↑GR, ↑nuclear accumulation of Nrf2	4 µM (topical)	4 days	Shan et al. (2016)

NADPH oxidase is another key ROS source that is enhanced by DADS. Rac2 activated the c-JNK pathway in DADS-induced apoptosis but did not activate the p38 pathway. NADPH oxidase, reactive oxygen species, and Rac2 played a role in the DADS-induced apoptosis of HL 60 human leukemia cells (Yi et al., 2010). The expression of DJ 1 proteins was significantly reduced in the cytoplasm when the HL 60 cells were exposed to 1.25 mg/L DADS over 8 h. However, the expression DJ 1 was greatly elevated in the nucleus fractions compared to the untreated controls. Following treatment with 5 mg/L and 10 mg/L of DADS, the expression levels of DJ 1 proteins were dramatically reduced in the mitochondria of the HL 60 cells. These findings revealed that exposing HL 60 cells to low dosages of DADS increases the translocation of the DJ one protein from the cytoplasm to the nucleus, suggesting that DJ one could be a cofactor binding protein or transcription factor in cell differentiation. The expression of DJ one in mitochondria may be connected to the induction of apoptosis in HL 60 cells that were exposed to moderate concentrations of DADS (Li et al., 2016). Calreticulin (CRT) played a significant role in human acute myeloid leukemia (AML) cell invasion and proliferation and is the subject of a significant amount of research. Yi et al. (Yi et al., 2016) found that CRT caused cell differentiation, proliferation, and invasion in DADS-treated HL 60 cells, as evidenced by the DADS-induced CRT downregulation across differentiated HL 60 cells. Following DADS-induced differentiation, CRT expression levels decreased while C/EBPα expression levels increased in the HL 60 cells. In severe combined immunodeficiency mice injected with HL 60 cells, DADS reduced tumor tissue growth *in vivo*, decreased levels of CRT, and increased C/EBPα. Furthermore, it was demonstrated that the DADS-mediated increased expression of C/EBPα and decreased expression of CRT expression resulted in an upregulation of reactive oxidant species. In an RNA immunoprecipitation experiment, CRT bound to C/EBPα mRNA, indicating that it controls C/EBPα mRNA degradation by conjugating with UGrich elements in the 3′ untranslated region (Sun et al., 2019). The DADS-induced arrest of the G2/M phase in HL-60 cells may be linked to the activation of p38 MAP kinase. Following the expression of phospho-Cdc2 and phospho-Cdc25B, DADS elevated the expression levels of phospho-p38 MAPK and activated the G2/M checkpoint when HL-60 cells were exposed to 20 µM/L of DADS over 12 h (Tan et al., 2004).

DADS dramatically inhibited the growth of HL-60 cells by suppressing the expression of VEGF mRNA and the generation of VEGF proteins in HL-60 cells, leading to anti-leukemic effects (Fan et al., 2006). As the concentration of DADS was increased, the number of K562 cells reduced significantly, and the form of some of the fixed K562 cells became irregular, resulting in a twisted membrane. The number of green spots in the stained cells increased as the concentration of DADS increased. The rate of autophagy in K562 cells increased steadily after 48 h of DADS culture. The groups that had been exposed to 20, 40, and 80 mg/L of DADS exhibited greater autophagy rates compared to the group of blank control, of which the group that had been exposed to 40 mg/L of DADS exhibited the highest autophagy rate. There was no discernable variation in the expression of ERK protein between groups, but the expression of p-ERK and LC3-II proteins increased as the concentration of DADS increased; a substantial change in the expression of the proteins of the 40 mg/L DADS group was also observed. DADS activated the MEK-ERK signaling pathway through the phosphorylation of ERK, which induced autophagy in the K562 cells (Ye and Yin, 2017).

#### 2.4.2 Lymphoma

Lymphomas refer to solid tumors found in the immune system. Hodgkin lymphomas account for 10% of all lymphomas. Lymphomas are relatively common and most clinicians will have encountered a lymphoma patient regardless of their specialty. Early diagnosis is critical since many lymphomas have excellent, and quite curative, therapies (Pinkerton, 2013). DADS inhibited the activity of telomerase through the transcriptional downregulation of hTERT, the catalytic subunit of telomerase, while keeping the expression of its RNA component unchanged. The suppression of the transcription factors Sp-1 and c-Myc by caspases and the cysteine protease, calpain, led to decreased DNA-binding efficiency at their relative binding sites on the hTERT promoter, culminating in apoptosis *via* the reduction of telomerase activity. Elevated Mad1 levels generated by DADS treatment may also lead to the creation of additional Max/Mad complexes that may interfere with the Max/Myc complex which binds the E-box of the hTERT promoter, thus transcriptionally decreasing the expression of hTERT (Table 2) (Dasgupta and Sengupta Bandyopadhyay, 2015).

### 2.5 Lung Cancer

Non-small cell lung cancer (NSCLC) is the single biggest reason for cancer-associated fatalities. Radiotherapy is still the primary treatment for NSCLC. However, ionizing radiation (IR) at low doses can cause invasion and migration (Xu et al., 2021). One study showed that IR significantly boosted A549 cell invasion and migration. A549 cells treated with 40 µM of DADS decreased the IR-induced invasion and migration while improving their radiosensitivity. Furthermore, IR-induced EMT was inhibited by 40 µM of DADS by suppressing the protein matrix, which is related to metalloproteinase-9 (MMP-9) and metalloproteinase-2 (MMP-2) generation, as well as the reduction of the mesenchymal marker N-cadherin and the elevation of the epithelial marker E-cadherin in A549 cells. In addition to this, the expression of Nrf2 signaling was shown to be inhibited by DADS. The IR-induced invasion and migration were suppressed by DADS by inhibiting the activation of Nrf2 signaling in A549 cells (Table 2) (Xu et al., 2021).

**FIGURE 1:**
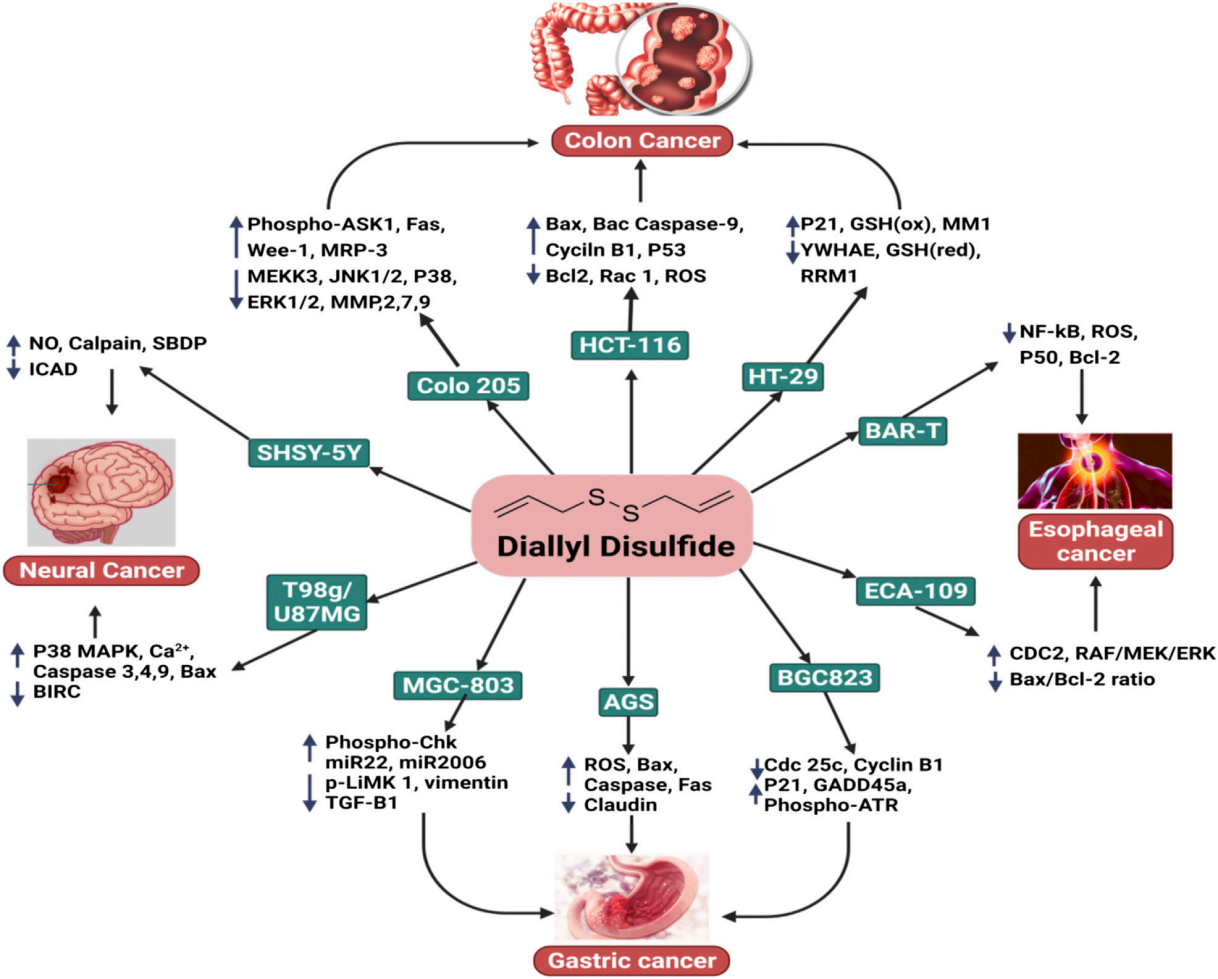
The anticancer activity of DADS with respect to colon cancer, esophageal cancer, gastric cancer, and neural cancer.

The EMT is critical to the process of malignant transformations, and fibronectin (FN), a component of the extracellular matrix, can induce metastasis and invasion. Due to the reduced function of gelatinase, DADS hindered the FN-induced migration and invasion of A549 cells. In DADS-treated A549 cells, cytokeratin-18 and E-cadherin, which are epithelial indicators, were elevated, while vimentin and N-cadherin, which are the mesenchymal markers, as well as factors of transcription, such as twist, snail, and slug, were downregulated. DADS inhibited the FN-induced nuclear translocation of catenin and glycogen synthase kinase-3 phosphorylation in A549 cells, as well as disorganized lymphoid enhancer factor/T-cell factor and homolog 2 activities (Figure 1) (Das and Sinha, 2019).

### 2.6 Neural Cancer

Neural cancers are a heterogeneous group of over 100 illnesses that cause considerable morbidity and mortality when combined. Glioblastoma multiform (GBM), the most common form of brain tumor, is almost always fatal, with a survival rate (5-year) of only 2%; current treatments only provide palliative relief. GBMs have a high degree of cellular heterogeneity, which may explain why patients’ nodular or regional patterns frequently advance or recur (Figure 1) (Lathia et al., 2011).

Due to its extensive cytoskeletal modification and particularly lethal effect on growing neuroblastoma cells, Aquilano et al. (2010) emphasized the use of DADS in cancer therapy. As the phosphorylation of Tau is strongly associated with the functions of the neuronal cytoskeleton, their investigations found Tau to be a novel target for the anti-cytoskeletal action of DADS and can be used to develop new ways of treating neuronal illnesses linked with Tau phosphatases and the impairment of hyperphosphorylation (Aquilano et al., 2010). DADS has been shown to suppress antiapoptotic factors while simultaneously inherently triggering a caspase cascade as well as activating calpain, resulting in the apoptosis of SH-SY5Y cells (Karmakar et al., 2007). DADS was also found to regulate nNOS, suggesting that nitric oxide plays a significant role in preventing the cytotoxicity caused by reactive oxygen species (Aquilano et al., 2007). DADS exhibited potential anti-glioma properties, especially with regard to their proliferation, while also inducing apoptosis in four distinct types of glioma cell lines that represented the different phases of the illness (Choromanska et al., 2020). DADS caused glioblastoma cells to die by forming ROS, increasing ER stress, lowering ∆Ψm, and activating apoptosis-inducing cysteine proteases and stress kinases (Table 2) (Das et al., 2007).

### 2.7 Skin Cancer

Skin cancer is a lethal illness and a major public health issue that has resulted in significant economic and human losses across the world (Didona et al., 2018). A variety of internal and environmental variables can aggravate the pathophysiology of skin cancer and worsen the illness (Siegel et al., 2018). The importance of the early identification and diagnosis of skin cancer cannot be overstated. The mortality rate of skin cancer has decreased dramatically because of improved screening procedures, early detection and diagnosis, and innovative treatment modalities (Table 2) (Casari et al., 2018).

The topical application of DADS reduced the prevalence and development of skin cancer in mice models. Shan et al. (2016) revealed that DADS decreased the occurrence and development of skin tumors in mice models in a dose-dependent manner. This mechanism was associated with the upregulation of antioxidant enzyme activity, including catalase, SOD, and glutathione peroxidase, as well as the nuclear accumulation of Nrf2. DADS also enhanced the endogenous link between Nrf2 and p21 and was critical in helping Keap-1 prevent the degradation of Nrf2 (Figure 1) (Shan et al., 2016).

### 2.8 Prostate Cancer

Prostate cancer is the second most frequent cancer in males after skin cancer, and yet its treatments have the highest success rates. An *in vitro* experiment was used to study the impact of DADS on growth factor signaling molecules (like insulin) that are involved in the proliferation and survival of the human prostate cancer cells (Arunkumar et al., 2012). It was found that DADS reduces the rate of survival of androgen-independent prostate cancer cells by modulating the expression of the IGF system, resulting in the inhibition of Akt phosphorylation, and consequently inhibiting cell cycle survival and progression by reducing the expression of NF-kB, cyclin D1, and antiapoptotic Bcl-2 molecules while enhancing the expression of proapoptotic signaling molecules (Bax and Bad) which trigger apoptosis (Arunkumar et al., 2012). Shin et al. (2010) investigated the anti-invasive potential of DADS in prostate cancer LNCaP cells; its mechanism involved the tightening of TJs and inhibiting matrix metalloproteinase activities. DADS inhibited the expression of claudin proteins, the important components of TJs, which are crucial for the selectivity and regulation of paracellular transport. In addition, the administration of DADS suppressed the activity of MMP-9 and MMP-2 in LNCaP cells in a dose-dependent manner; this was also associated with a decrease in the expression of proteins and mRNA (Table 2) (Shin et al., 2010).

**FIGURE 2:**
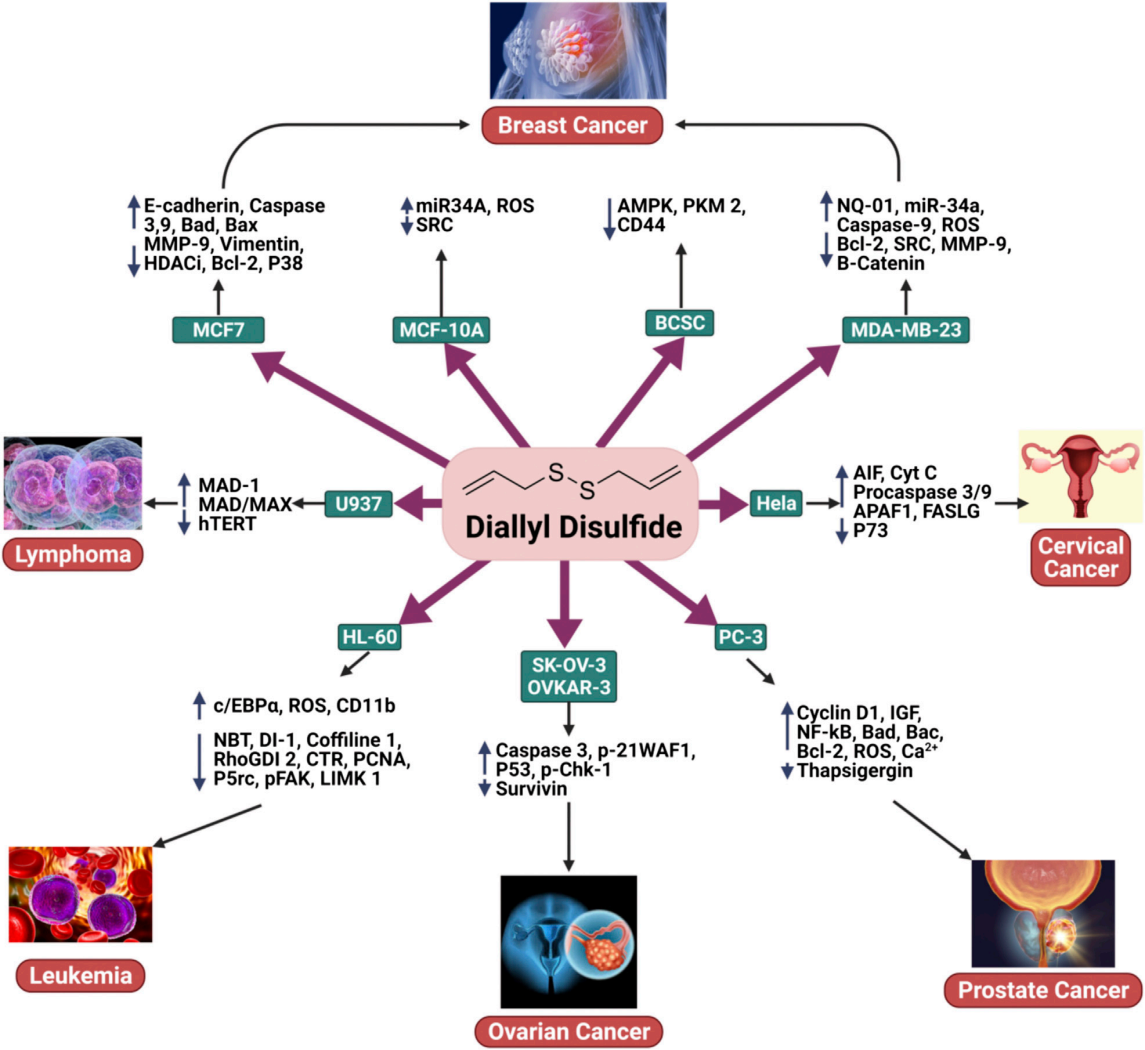
The anticancer activity of DADS in breast cancer, ovarian cancer, cervical cancer, prostate cancer, leukemia, and lymphoma.

Chen et al. studied the impacts of DADS on Ca^2+^ viability and mobility in human prostate cancer PC3 cells. 500 μM of DADS caused apoptosis in a mechanism that was independent of Ca^2+^. Annexin V/pi staining revealed that concentrations of both 10 and 500 μM of DADS induced apoptosis. DADS also boosted the formation of ROS. DADS caused an increase in Ca^2+^ in PC3 cells by inducing phospholipase C-independent Ca^2+^ release from the endoplasmic reticulum as well as the influx of Ca^2+^ *via* phospholipase A2 sensitive channels. In summary, DADS induced Ca^2+^-independent apoptosis, Ca^2+^-dependent cell death, and the generation of ROS (Figure 2) (Chen et al., 2011).

DADS significantly suppressed the development of human prostate cancer DU145 cells by inducing apoptosis. Apoptosis was accompanied by the modulation of Bcl-2 and the inhibitor of apoptosis proteins (IAP) family of proteins, the depolarization of the mitochondrial membrane potential (MMP, *ΔΨm*), and proteolytic activation of caspases. DADS boosted the expression of Fas ligand (FasL) and death receptor 4 (DR4) proteins while decreasing the number of intact Bid proteins. Furthermore, DADS stimulated the phosphorylation of mitogen-activated protein kinases (MAPKs) such as extracellular-signal regulating kinase (ERK), p38 MAPK, and c-Jun N-terminal kinase (JNK). SP600125, an inhibitor of JNK, greatly inhibited DADS-induced apoptosis, but p38, MAPK (SB203580), and ERK (PD98059) inhibitors did not produce the same effect. DADS-induced apoptosis was followed by the inactivation of phosphatidylinositol 3-kinase (PI3K)/Akt and the inhibition of PI3K. In addition, LY29004 dramatically boosted DADS-induced cell death (Shin et al., 2012).

The growth and progression of breast and prostate cancers have been associated with NF-κB activation. DADS had an IC-50 value of 40 µM whereas MDA-MB-231 and MCF-7 cells had IC-50 values of 6 and 4 µM, respectively. PC3 cells were administered with DADS/quercetin, which significantly reduced the expression of NF-κB, IKKα, and IKKβ. This suggests that DADS or quercetin blocks the expression of nuclear factor kappa-B in androgen-independent prostate cancer cells (Arunakaran et al., 2013).

## 3 Bioavailability and Pharmacokinetic Profile of Diallyl Disulfide

A comprehensive investigation of DADS pharmacokinetics profiles was conducted by orally administering a 200 mg/kg dose to rats. Allylmethyl sulfide (AMS), allylmercaptan (AM), allyl methyl sulfone, and allyl methyl sulfoxide were recognized as DADS metabolites in the plasma, stomach, urine, and liver of rats (Zhang et al., 2020). When the prepared garlic was consumed, the alliinase enzyme was activated, accelerating the transformation of alliin to allicin, an unstable metabolite. The decomposition of allicin leads to the formation of organic diallyl polysulfides such as diallyl sulfide (DAS) and DADS (Bradley et al., 2016; Yamaguchi and Kumagai, 2019; He et al., 2021). In the human body, DADS degrades quickly, metabolizing into different metabolites, such as diallyl thiosulphinate (DADSO), allylmercaptan (AM), allylmethyl sulfoxide (AMSO), allylmethyl sulfide (AMS), and allylmethyl sulfone (AMSO2). The presence of DADS has been identified in the breath of human subjects following the consumption of garlic. DADS could not be identified in the urine of a volunteer after the oral intake of 1–3 g of garlic powder; this was because DADS cannot easily reach μM-level concentrations *in vivo* (Alma et al., 2014; He et al., 2021). Similarly, allicin is rapidly removed from circulation after intravenous injections as it is converted into secondary metabolites such as DADS, 2-ethenyl-4H-1,3-dithiin, and E-ajoene (Jacob and Anwar, 2008; Ansary et al., 2020).

For enteric tablets, the allicin, which would eventually metabolized to DADS, various bioavailability were found ranging from 36% to 104%. This was reduced to 22%–57% when consumed with a high-protein meal. Nonenteric tablets gave high bioavailability (80%–111%), while garlic powder capsules gave 26%–109% bioavailability, whatever the meal type is Allicin rapidly disappears from circulation after iv injection, suggesting that it is transformed into secondary products. (Zhang et al., 2020).

Yamaguchi and Kumagai, (2019) found in their animal studies that alliin was absorbed primarily from the intestine *via* the amino acid transporter for cysteine. The split products of alliin appeared in the circulation 20 min after the administration of alliin by gastric intubation. These investigators concluded that splitting of alliin would take place in the intestinal cells by the action of an alliinase (or C-S-lyase)-like enzyme. The sulfides derived from allicin are known to be absorbed very fast from the intestine, which can be attributed to chemopreventive potential of this molecule (Ariga and Seki, 2006). The bioavailability of OSCs is high in animal subjects treated with aqueous garlic extracts (98% in liver, 103% in blood plasma, and 87% in kidneys for rats, mice, and dogs, respectively). It was estimated that 1 g of ingested garlic will biosynthesize 2.5 mg of allicin, 60 μg of SAC, 1,000 ± 100 μg of DATS, and 570 ± 40 μg of DADS (Gao et al., 2013).

The bioactivity of Allium is mainly attributed to the allyl derivatives that showed *in vivo* anticarcinogenic activity in various tissues. For instance, in garlic extracts, OSCs are mainly found in allyl and methyl forms (Thompson, 2014; Ramirez et al., 2017). These clearly demonsrates that the significant amount of these molecules can contribute to anticancer properties of DADS.

DADS was determined to have a half-life of less than 1 h in isolated rat livers. Four metabolites of DADS, AMS, AM, AMSO2, and AMSO, were determined to have half-lives (T_1/2_) of 6.78, 4.39, 8.64, and 7.16 h, respectively. AMS, AM, AMSO2, and AMSO were found to have the peak concentrations (C_max_) of 8, 8, 1,440, and 376 µM, respectively, suggesting that it is possible to achieve effective therapeutic concentrations of these active metabolites. DADS was shown to be quickly absorbed, with the peak concentrations observed 90 min after it was administered. After 2 h, 70% of the radioactivity was still detectable in the cytosol of liver cells, 80% of which was metabolized into sulfides while only 8% as remained as 35S-DADS. Within 2–3 h, DATS, AMDS, DADS, and DAS reached their maxima while the rate of increase of the other compounds was slower (Haina Wang, 2013).

The RBCs of humans convert the organic polysulfides that are derived from garlic into H_2_S, an endogenous cardio-protective vascular cell-signaling molecule. Increasing numbers of sulfur-tethering atoms and substituents of allyl accelerate the production of H_2_S from the organic polysulfides. Hydropolysulfide (RSnH) is a critical intermediate in the production of H_2_S; RSnH is formed when polysulfides that are allyl-substituted undergo nucleophilic substitution at the α-carbon of the allyl substituent. Nucleophilic substitution takes place at the sulfur atom of the organic polysulfides, yielding H_2_S and RS_n_H. H_2_S is also released when the intact aorta rings process the garlic-derived organic polysulfides under physiologically relevant oxygen levels (Benavides et al., 2007).

The behavior of drug release in different pH environments, such as the intracellular lysosome (pH 4.5) and the cellular exterior (pH 7.4), was investigated. The profile of the regulated release of DADS from solid lipid nanoparticles (SNL) was examined. Under these conditions, a greater DADS release rate was observed at lower pHs. Due to the presence of the pair of sulfide groups, DADS acts as both an alkaline and a weak acid and is more soluble at lower pHs. As a result, DADS encapsulated within SLN were more likely to be released at lower pH. Drug release in a more favorable acidic environment would result in a greater rate of release of DADS to the tumor cells; this is especially important in cell lines that are resistant, increasing the therapeutic potential of the delivery system (Talluri et al., 2017; Siddhartha et al., 2018).

## 4 Clinical Studies

According to the FDA’s proof-based assessment procedure for scientific health appraisal, there is no clear evidence that links garlic to decreased risks of lung, breast, or gastric cancer. (Rivlin, 2009). Even though almost all experiments were observational, the number of investigations that were deemed to be scientifically relevant to this assessment was comparatively small. In addition, the number of subjects involved was relatively small, and no conclusive proof regarding a link between garlic consumption and esophageal, oral, colon, ovarian, prostate, renal, and laryngeal cell cancers has been recorded. Consequently, the relationship between cancer risk reduction and garlic remains unknown (Zuniga et al., 2019). Garlic appears to assist with a wide range of cancer symptoms, including those associated with pancreatic, lung, colon, gastric, colorectal, and breast cancers. In this context, a potential personalized diet with supplementary foods, including beneficial phytochemicals such as allyl sulfur compounds and allicin, could be a potential alleviative treatment. Patients in remission or undergoing therapy have been administered high dosages of allicin antioxidants (Table 3) (Ansary et al., 2020).

**TABLE 3:** Clinical studies on garlic constituents in cancer prevention and intervention.

Subjects	Types of study	Population of study	No. of patients	Intervention	Key findings	References
Breast cancer patient	Randomized intervention trial	United States	153	39.0%–69.5% garlic diet	Improved adherence to a Mediterranean style	Zuniga et al. (2019)
Individuals with gastric lesion	Factorial, double-blind, placebo-controlled trial	China	4,326	AGE 400 mg	Reduction of burden of gastric cancer in high risk areas	Gail and You, (2006)
Gastric cancer patient	Factorial, double-blind, placebo-controlled trial	China	3,411	4 capsules/day	Elevated concentration of serum folate	Wang et al. (2009)
Colorectal cancer patient	Comparison based study	Germany	57,560	One bulb/day	Condensed colorectal adenoma risk	Dreher, (2018)
Healthy adults	Case-control	China	966 men and 700 women	—	Condensed colorectal adenoma risk	Wu et al. (2019)
Lung cancer patient and healthy adults	Case-control	China	5,967	garlic compounds 33.4 g per week	Chemopreventive effect	Jin et al. (2013)
Hematological patients	Double-blind, placebo-controlled trial	Israel	95	900 mg/day	No significant effects in the entire cohort	Gatt et al. (2015)
Healthy adults	Randomized crossover feeding trial	United States	17	—	Activated genes correlated to apoptosis, immunity, and xenobiotic metabolism	Charron et al. (2015)

A 6-month eating plan that followed a Mediterranean-style diet was found to increase the intake of anti-inflammatory ingredients such as garlic among the survivors of breast cancer (Zuniga et al., 2019). Another experiment revealed that taking 200 mg capsules or 1 mg of garlic oil twice a day decreased progressive gastric lesions (You et al., 2006). Similar outcomes were observed when the same doses of garlic supplementations were administered over 7.3 years. In addition, long-term garlic intake, such as garlic pills or garlic mixed with vitamins reduced cancer risk (Ma et al., 2012), mortality rate (Li et al., 2019), and precancerous gastric lesions (Gail and You, 2006). A garlic treatment of two capsules twice a day for 7.3 years improved mild folate insufficiency and enhanced serum folate in individuals who were experiencing gastric lesions in rural Chinese communities (Wang et al., 2009). In addition, consuming 3.65 kg of garlic supplements per year for 2 years was linked to a lower incidence of colorectal adenoma, a precursor to colorectal cancer (CRC) (Table 3) (Jin et al., 2013; Gatt et al., 2015; Dreher, 2018; Wu et al., 2019). Several theories have been proposed to explain the chemopreventive benefits of garlic, including the suppression of the formation of DNA adducts, the inhibition of mutagenesis by limiting metabolism, the scavenging of free radicals, or the lowering of cell growth and tumor development (Jin et al., 2013; Charron et al., 2015). In another study, cancer patients were asked to follow either the remission support diet (RD; for the patients in remission) or the treatment support diet (TD; for the patients undergoing chemotherapy) over 3–9 weeks. The diets were low in fat and glucose and high in plant proteins; however, the TD group had an extra 0.5 protein servings. Additional quantities of tomato, rice bran, garlic, kale, pineapples, onion, blueberry, turmeric powder, and/or shiitake were included in daily meals based on clinical studies. The TD had a higher estimated daily consumption of quercetin, plant fat, allicin, onion, protein, and garlic than the RD. Both groups experienced an elevated consumption of vitamin A, C, and E, as well as a decreased consumption of the D-dimer relative to baseline diets. TD showed a greater impulse in cytotoxicity and increased albumin while RD showed reduced D-ROMS (Lee et al., 2015).

## 5 Conclusion and Future Recommendations

Garlic, a widely consumed spice of the genus *Allium*, has been found to contain various organosulfur components. DADS has received attention in cancer prevention research as a natural product with potent anticancer properties. This review aims to provide an inclusive evaluation of clinical and preclinical research on the chemopreventive and anticancer effects of DADS. Toxicity and pharmacokinetic investigations of DADS were also included. The potential of this phytochemical as an anti-cancer agent has been supported by many *in vitro* and *in vivo* investigations. Pharmacokinetic investigations revealed that this chemical has high bioavailability in a variety of tissues. Injections are the most common delivery method in animal experiments, but clinical experiments have focused on oral ingestion. Future animal studies should more efficiently mimic the conditions of clinical trials conditions to obtain a better understanding of the actual anticancer effectiveness of DADS. The bulk of the literature discussed in this review focuses on preclinical investigations; however, it also covers clinical tests conducted on DADS and its biogenic precursors. These investigations proposed several mechanistic pathways for DADS’ anticancer effects, such as invasion, migration, metastasis, cell cycle arrest, oxidative stress, and cell death. Many *in vitro* investigations have shown that DADS induces several distinct anticancer activities across a variety of cancer subtypes. More *in vivo* research is required to provide support for these mechanisms. Garlic was also used in several trials; however, this review recommends concentrating on DADS due to its potency. Pure DADS should be studied in more detail to completely understand its anticancer characteristics, especially since several investigations have proposed conflicting mechanisms. More *in vivo* studies must be conducted to elucidate the true mechanisms and the target biomolecules of DADS, while also identifying biomarkers that can measure the effectiveness of DADS in anticancer therapy. The literature suggests that DADS could be a promising agent for future natural chemotherapy and that it has significant potential as a safe and efficacious natural remedy to cancer.

## References

[B1] Agassi S. F. T. Yeh T. M. Chang C. D. Hsu J. L. Shih W. L. (2020). Potentiation of differentiation and apoptosis in a human promyelocytic leukemia cell line by garlic essential oil and its organosulfur compounds. Anticancer Res. 40, 6345–6354. 10.21873/anticanres.14655 33109572

[B2] Ahmed O. Ahmed R. (2015). Anti-proliferative and apoptotic efficacy of diallyl disulfide on Ehrlich ascites carcinoma. Hepatoma Res. 1, 67. 10.4103/2394-5079.157602

[B3] Alma E. Eken A. Ercil H. Yelsel K. Daglioglu N. (2014). The effect of garlic powder on human urinary cytokine excretion. Urol. J. 11, 1308–1315. 24595942

[B4] Altonsy M. O. Habib T. N. Andrews S. C. (2012). Diallyl disulfide-induced apoptosis in a breast-cancer cell line (MCF-7) may be caused by inhibition of histone deacetylation. Nutr. Cancer 64, 1251–1260. 10.1080/01635581.2012.721156 23163853

[B5] An X. Zhang X. Yao H. Li H. Ren J. (2015). Effects of diallyl disulfide in elephant garlic extract on breast cancer cell apoptosis in mitochondrial pathway. J. Food Nutr. Res. (Newark). 3, 196–201. 10.12691/jfnr-3-3-11

[B6] Ansari I. A. Ahmad A. Imran M. A. Saeed M. Ahmad I. (2020). Organosulphur compounds induce apoptosis and cell cycle arrest in cervical cancer cells via downregulation of HPV E6 and E7 oncogenes. Anticancer. Agents Med. Chem. 21, 393–405. 10.2174/1871520620999200818154456 32819236

[B7] Ansary J. Forbes-Hernández T. Y. Gil E. Cianciosi D. Zhang J. Elexpuru-Zabaleta M. (2020). Potential health benefit of garlic based on human intervention studies: A brief overview. Antioxidants 9, E619–E635. 10.3390/antiox9070619 32679751 PMC7402177

[B8] Anwanwan D. Singh S. K. Singh S. Saikam V. Singh R. (2020). Challenges in liver cancer and possible treatment approaches. Biochim. Biophys. Acta. Rev. Cancer, 1873, 188314. 10.1016/j.bbcan.2019.188314 31682895 PMC6981221

[B9] Aquilano K. Filomeni G. Baldelli S. Piccirillo S. De Martino A. Rotilio G. (2007). Neuronal nitric oxide synthase protects neuroblastoma cells from oxidative stress mediated by garlic derivatives. J. Neurochem. 101, 1327–1337. 10.1111/j.1471-4159.2006.04431.x 17298386

[B10] Aquilano K. Vigilanza P. Filomeni G. Rotilio G. Ciriolo M. R. (2010). Tau dephosphorylation and microfilaments disruption are upstream events of the anti-proliferative effects of DADS in SH-SY5Y cells. J. Cell. Mol. Med. 14, 564–577. 10.1111/j.1582-4934.2008.00588.x 19040422 PMC3823456

[B11] Ariga T. Seki T. (2006). Antithrombotic and anticancer effects of garlic-derived sulfur compounds: A review. BioFactors 26, 93–103. 10.1002/biof.5520260201 16823096

[B12] Arunakaran J. Arunkumar R. Elumalai P. Senthilkumar K. (2013). Impact of quercetin, diallyl disulfide and nimbolide on the regulation of nuclear factor kappa B expression in prostate and breast cancer cell lines. Nat. Prod. Chem. Res. 1. 10.4172/2329-6836.1000115

[B13] Arunkumar R. Sharmila G. Elumalai P. Senthilkumar K. Banudevi S. Gunadharini D. N. (2012). Effect of diallyl disulfide on insulin-like growth factor signaling molecules involved in cell survival and proliferation of human prostate cancer cells *in vitro* and *in silico* approach through docking analysis. Phytomedicine. 19, 912–923. 10.1016/j.phymed.2012.04.009 22739413

[B14] Bauer D. Mazzio E. Soliman K. F. Taka E. Oriaku E. Womble T. (2014). Diallyl disulfide inhibits TNFα-induced CCL2 release by MDA-MB-231 cells. Anticancer Res. 34, 2763–2770. 24922637 PMC4135704

[B15] Bauer D. Redmon N. Mazzio E. Taka E. Reuben J. S. Day A. (2015). Diallyl disulfide inhibits TNFα induced CCL2 release through MAPK/ERK and NF-Kappa-B signaling. Cytokine 75, 117–126. 10.1016/j.cyto.2014.12.007 26100848 PMC4532635

[B16] Benavides G. A. Squadrito G. L. Mills R. W. Patel H. D. Isbell T. S. Patel R. P. (2007). Hydrogen sulfide mediates the vasoactivity of garlic. Proc. Natl. Acad. Sci. U. S. A. 104, 17977–17982. 10.1073/pnas.0705710104 17951430 PMC2084282

[B17] Bigby J. (1988). Harrison’s principles of internal medicine. Arch. Dermatol. 124, 287. 10.1001/archderm.1988.01670020093028

[B18] Bo S. Hui H. Li W. Hui L. Hong X. Lin D. (2014). Chk1, but not Chk2, is responsible for G2/M phase arrest induced by diallyl disulfide in human gastric cancer BGC823 cells. Food Chem. Toxicol. 68, 61–70. 10.1016/j.fct.2014.03.007 24650757

[B19] Bo S. Jian S. Ying Z. Fang L. Hong X. Yan-Hua M. (2016). Diallyl disulfide suppresses epithelial-mesenchymal transition, invasion and proliferation by downregulation of LIMK1 in gastric cancer. Oncotarget 7, 10498–10512. 10.18632/oncotarget.7252 26871290 PMC4891135

[B20] Bradley J. M. Organ C. L. Lefer D. J. (2016). Garlic-derived organic polysulfides and myocardial protection. J. Nutr. 146, 403S–409S. 10.3945/jn.114.208066 26764335 PMC4725427

[B21] Brennan S. F. Cantwell M. M. Cardwell C. R. Velentzis L. S. Woodside J. V. (2010). Dietary patterns and breast cancer risk: A systematic review and meta-analysis. Am. J. Clin. Nutr. 91, 1294–1302. 10.3945/ajcn.2009.28796 20219961

[B22] Casari A. Chester J. Pellacani G. (2018). Actinic keratosis and non-invasive diagnostic techniques: An update. Biomedicines 6, E8. 10.3390/biomedicines6010008 29316678 PMC5874665

[B23] Charron C. S. Dawson H. D. Albaugh G. P. Solverson P. M. Vinyard B. T. Solano-Aguilar G. I. (2015). A single meal containing raw, crushed garlic influences expression of immunity- and cancer-related genes in whole blood of humans. J. Nutr. 145, 2448–2455. 10.3945/jn.115.215392 26423732 PMC4620724

[B24] Chen C. Y. Huang C. F. Tseng Y. T. Kuo S. Y. (2012). Diallyl disulfide induces Ca 2+ mobilization in human colon cancer cell line SW480. Arch. Toxicol. 86, 231–238. 10.1007/s00204-011-0748-4 21879349

[B25] Chen W. C. Hsu S. S. Chou C. T. Kuo C. C. Huang J. K. Fang Y. C. (2011). Effect of diallyl disulfide on Ca2+ movement and viability in PC3 human prostate cancer cells. Toxicol. Vitro. 25, 636–643. 10.1016/j.tiv.2010.12.015 21232596

[B26] Chen X. X. Liu X. W. Zhou Z. G. Chen X. Y. Li L. D. Xiong T. (2016). Diallyl disulfide inhibits invasion and metastasis of MCF-7 breast cancer cells *in vitro* by down-regulating p38 activity. Nan Fang. Yi Ke Da Xue Xue Bao 36, 814–818. 27320884

[B27] Choromanska A. Kulbacka J. Saczko J. Surowiak P. (2020). Effect of diallyl disulfide and garlic oil on different human astrocytoma cell lines. Biomed. Rep. 13, 32–36. 10.3892/br.2020.1339 32802329 PMC7412714

[B28] Comprehensive N. Network C. (2014). Esophageal and esophagogastric junction.

[B29] Dabrowski A. Abramowicz K. Zinkiewicz K. (1998). Epidemiology of esophageal cancer. Pol. Merkur. Lek. 5, 145–172. 10.1007/978-1-4684-2442-3_7 10101462

[B30] Das A. Banik N. L. Ray S. K. (2007). Garlic compounds generate reactive oxygen species leading to activation of stress kinases and cysteine proteases for apoptosis in human glioblastoma T98G and U87MG cells. Cancer 110, 1083–1095. 10.1002/cncr.22888 17647244

[B31] Das B. Sinha D. (2019). Diallyl disulphide suppresses the cannonical Wnt signaling pathway and reverses the fibronectin-induced epithelial mesenchymal transition of A549 lung cancer cells. Food Funct. 10, 191–202. 10.1039/c8fo00246k 30516195

[B32] Dasgupta P. Sengupta Bandyopadhyay S. (2015). Role of diallyl disulfide-mediated cleavage of c-Myc and Sp-1 in the regulation of telomerase activity in human lymphoma cell line U937. Nutrition 31, 1031–1037. 10.1016/j.nut.2015.02.016 26059379

[B33] De Greef D. Barton E. M. Sandberg E. N. Croley C. R. Pumarol J. Wong T. L. (2021). Anticancer potential of garlic and its bioactive constituents: A systematic and comprehensive review. Semin. Cancer Biol. 73, 219–264. 10.1016/j.semcancer.2020.11.020 33301861

[B34] Deschler B. Lübbert M. (2008). Acute myeloid leukemia: Epidemiology and etiology. Acute Leuk., 47–56. 10.1007/978-3-540-72304-2_3 17019734

[B35] Di C. Sun C. Li H. Si J. Zhang H. Han L. (2015). Diallyl disulfide enhances carbon ion beams- induced apoptotic cell death in cervical cancer cells through regulating Tap73/ΔNp73. Cell Cycle 14, 3725–3733. 10.1080/15384101.2015.1104438 26505313 PMC4825711

[B36] Didona D. Paolino G. Bottoni U. Cantisani C. (2018). Non melanoma skin cancer pathogenesis overview. Biomedicines 6. 10.3390/biomedicines6010006 PMC5874663 29301290

[B37] Dixon K. Kopras E. (2004). Genetic alterations and DNA repair in human carcinogenesis. Semin. Cancer Biol. 14, 441–448. 10.1016/j.semcancer.2004.06.007 15489137

[B38] Dreher M. L. (2018). Dietary patterns, whole plant foods, nutrients and phytochemicals in colorectal cancer prevention and management. Diet. Patterns Whole Plant Foods Aging Dis., 521–555. 10.1007/978-3-319-59180-3_19

[B39] Druesne N. Pagniez A. Mayeur C. Thomas M. Cherbuy C. Duée P. H. (2004). Diallyl disulfide (DADS) increases histone acetylation and p21waf1/cip1 expression in human colon tumor cell lines. Carcinogenesis 25, 1227–1236. 10.1093/carcin/bgh123 14976134

[B40] Druesne-Pecollo N. Chaumontet C. Latino-Martel P. (2008). Diallyl disulfide increases histone acetylation in colon cells *in vitro* and *in vivo* . Nutr. Rev. 66, S39–S41. 10.1111/j.1753-4887.2008.00066.x 18673488

[B41] Elmore S. (2007). Apoptosis: A review of programmed cell death. Toxicol. Pathol. 35, 495–516. 10.1080/01926230701320337 17562483 PMC2117903

[B42] Elumalai P. Gunadharini D. N. Senthilkumar K. Banudevi S. Arunkumar R. Benson C. S. (2012). Induction of apoptosis in human breast cancer cells by nimbolide through extrinsic and intrinsic pathway. Toxicol. Lett. 215, 131–142. 10.1016/j.toxlet.2012.10.008 23089555

[B43] Fan Z. L. Qi Z. H. Xie Y. (2006). Effect of diallyl disulfide on the expression and secretion of VEGF in HL-60 cells. Zhonghua Xue Ye Xue Za Zhi 27, 626–629. 17278431

[B44] Feller L. L. Khammissa R. R. A. G. Kramer B. B. Lemmer J. J. (2013). Oral squamous cell carcinoma in relation to field precancerisation: Pathobiology. Cancer Cell Int. 13, 31. 10.1186/1475-2867-13-31 23552362 PMC3626548

[B45] Feng C. Luo Y. Nian Y. Liu D. Yin X. Wu J. (2017). Diallyl disulfide suppresses the inflammation and apoptosis resistance induced by DCA through ROS and the NF-κB signaling pathway in human barrett’s epithelial cells. Inflammation 40, 818–831. 10.1007/s10753-017-0526-4 28197857

[B46] Fleischauer A. T. Arab L. (2001). Garlic and cancer: A critical review of the epidemiologic literature. J. Nutr. 131, 1032S–1040S. 10.1093/jn/131.3.1032s 11238811

[B47] Fulda S. Debatin K. M. (2006). Extrinsic versus intrinsic apoptosis pathways in anticancer chemotherapy. Oncogene 25, 4798–4811. 10.1038/sj.onc.1209608 16892092

[B48] Gail M. H. You W. C. (2006). A factorial trial including garlic supplements assesses effect in reducing precancerous gastric lesions. J. Nutr. 136, 813S–815S. 10.1093/jn/136.3.813s 16484571

[B49] Gao S. Basu S. Yang G. Deb A. Hu M. (2013). Oral bioavailability challenges of natural products used in cancer chemoprevention. Prog. Chem. 25, 1553–1574.

[B50] Gatt M. E. Strahilevitz J. Sharon N. Lavie D. Goldschmidt N. Kalish Y. (2015). A randomized controlled study to determine the efficacy of garlic compounds in patients with hematological malignancies at risk for chemotherapy-related febrile neutropenia. Integr. Cancer Ther. 14, 428–435. 10.1177/1534735415588928 26036623

[B51] Globocan M. (2020). New Global Cancer, 1.

[B52] Greenwell M. Rahman P. K. S. M. (2015). Medicinal plants: Their use in anticancer treatment. Int. J. Pharm. Sci. Res. 6, 4103–4112. 10.13040/IJPSR.0975-8232.6(10).4103-12 26594645 PMC4650206

[B53] Haberkorn U. (2007). What is cancer? Adv. Nucl. Oncol. 2007, 1–16. 10.3109/9781420091380-2

[B54] Haina Wang X. J. (2013). Drug metabolism and pharmacokinetics of organosulfur compounds from garlic. J. Drug Metab. Toxicol. 04. 10.4172/2157-7609.1000159

[B55] Hasan H. F. Abdel-Hamid G. R. Ebrahim S. I. (2020). Antioxidant and anti-inflammatory effects of diallyl disulfide on hepatotoxicity induced by cyclophosphamide in rats. Nat. Prod. Commun. 15, 1934578X2096908. 10.1177/1934578X20969083

[B56] He H. Ma Y. Huang H. Huang C. Chen Z. Chen D. (2021). A comprehensive understanding about the pharmacological effect of diallyl disulfide other than its anti-carcinogenic activities. Eur. J. Pharmacol. 893, 173803. 10.1016/j.ejphar.2020.173803 33359648

[B57] Huang J. Yang B. Xiang T. Peng W. Qiu Z. Wan J. (2015). Diallyl disulfide inhibits growth and metastatic potential of human triple-negative breast cancer cells through inactivation of the β-catenin signaling pathway. Mol. Nutr. Food Res. 59, 1063–1075. 10.1002/mnfr.201400668 25755089

[B58] Huang Y. S. Xie N. Su Q. Su J. Huang C. Liao Q. J. (2011). Diallyl disulfide inhibits the proliferation of HT-29 human colon cancer cells by inducing differentially expressed genes. Mol. Med. Rep. 4, 553–559. 10.3892/mmr.2011.453 21468607

[B59] Islam M. R. Islam F. Nafady M. H. Akter M. Mitra S. Das R. (2022). Natural small molecules in breast cancer treatment: Understandings from a therapeutic viewpoint. Molecules 27, 2165. 10.3390/molecules27072165 35408561 PMC9000328

[B60] Jacob C. Anwar A. (2008). The chemistry behind redox regulation with a focus on sulphur redox systems. Physiol. Plant. 133, 469–480. 10.1111/j.1399-3054.2008.01080.x 18346080

[B61] Jin Z. Y. Wu M. Han R. Q. Zhang X. F. Wang X. S. Liu A. M. (2013). Raw garlic consumption as a protective factor for lung cancer, a population-based case-control study in a Chinese population. Cancer Prev. Res. 6, 711–718. 10.1158/1940-6207.CAPR-13-0015 PMC3718302 23658367

[B62] Jo H. J. Song J. D. Kim K. M. Cho Y. H. Kim K. H. Park Y. C. (2008). Diallyl disulfide induces reversible G2/M phase arrest on a p53-independent mechanism in human colon cancer HCT-116 cells. Oncol. Rep. 19, 275–280. 10.3892/or.19.1.275 18097607

[B63] Kamangar F. Chow W. H. Abnet C. Dawsey M. (2009). Environmental causes of esophageal cancer. Gastroenterol. Clin. North Am. 38, 27–57. 10.1016/j.gtc.2009.01.004 19327566 PMC2685172

[B64] Karmakar S. Banik N. L. Patel S. J. Ray S. K. (2007). Garlic compounds induced calpain and intrinsic caspase cascade for apoptosis in human malignant neuroblastoma SH-SY5Y cells. Apoptosis. 12, 671–684. 10.1007/s10495-006-0024-x 17219050

[B65] Kim H. J. Kang S. Kim D. Y. You S. Park D. Oh S. C. (2019). Diallyl disulfide (DADS) boosts TRAIL-Mediated apoptosis in colorectal cancer cells by inhibiting Bcl-2. Food Chem. Toxicol. 125, 354–360. 10.1016/j.fct.2019.01.023 30677442

[B66] Kim S. H. Lee I. C. Baek H. S. Shin I. S. Moon C. Bae C. S. (2014). Mechanism for the protective effect of diallyl disulfide against cyclophosphamide acute urotoxicity in rats. Food Chem. Toxicol. 64, 110–118. 10.1016/j.fct.2013.11.023 24291451

[B67] Ko J. W. Park S. H. Shin N. R. Shin J. Y. Kim J. W. Shin I. S. (2017). Protective effect and mechanism of action of diallyl disulfide against acetaminophen-induced acute hepatotoxicity. Food Chem. Toxicol. 109, 28–37. 10.1016/j.fct.2017.08.029 28847761

[B68] Lai K. C. Hsu S. C. Kuo C. L. Yang J. S. Ma C. Y. Lu H. F. (2013). Diallyl sulfide, diallyl disulfide, and diallyl trisulfide inhibit migration and invasion in human colon cancer colo 205 cells through the inhibition of matrix metalloproteinase-2, -7, and -9 expressions. Environ. Toxicol. 28, 479–488. 10.1002/tox.20737 21695758

[B69] Lai K. C. Kuo C. L. Ho H. C. Yang J. S. Ma C. Y. Lu H. F. (2012). Diallyl sulfide, diallyl disulfide and diallyl trisulfide affect drug resistant gene expression in colo 205 human colon cancer cells *in vitro* and *in vivo* . Phytomedicine. 19, 625–630. 10.1016/j.phymed.2012.02.004 22397993

[B70] Lathia J. D. Heddleston J. M. Venere M. Rich J. N. (2011). Deadly teamwork: Neural cancer stem cells and the tumor microenvironment. Cell Stem Cell 8, 482–485. 10.1016/j.stem.2011.04.013 21549324 PMC3494093

[B71] Lee G. Y. Lee J. J. Lee S. M. (2015). Antioxidant and anticoagulant status were improved by personalized dietary intervention based on biochemical and clinical parameters in cancer patients. Nutr. Cancer 67, 1083–1092. 10.1080/01635581.2015.1073754 26333154

[B72] Lee I. C. Kim S. H. Baek H. S. Moon C. Kang S. S. Kim S. H. (2014). The involvement of Nrf2 in the protective effects of diallyl disulfide on carbon tetrachloride-induced hepatic oxidative damage and inflammatory response in rats. Food Chem. Toxicol. 63, 174–185. 10.1016/j.fct.2013.11.006 24246655

[B73] Lee J. E. Lee R. A. Kim K. H. Lee J. H. (2011a). Induction of apoptosis with diallyl disulfide in AGS gastric cancer cell line. J. Korean Surg. Soc. 81, 85–95. 10.4174/jkss.2011.81.2.85 22066106 PMC3204569

[B74] Lee S. T. Li Z. Wu Z. Aau M. Guan P. Karuturi R. K. M. (2011b). Context-specific regulation of NF-κB target gene expression by EZH2 in breast cancers. Mol. Cell 43, 798–810. 10.1016/j.molcel.2011.08.011 21884980

[B75] Lei H. Hemminki K. Johansson R. Altieri A. Enquist K. Henriksson R. (2008). PAI-1 -675 4G/5G polymorphism as a prognostic biomarker in breast cancer. Breast Cancer Res. Treat. 109, 165–175. 10.1007/s10549-007-9635-3 17616807

[B76] Li Q. Tang Y. Qin J. Yi L. Yang Y. Wang J. (2016). Subcellular localization of DJ-1 in human HL-60 leukemia cells in response to diallyl disulfide treatment. Mol. Med. Rep. 14, 4666–4672. 10.3892/mmr.2016.5831 27748821 PMC5102037

[B77] Li W. Q. Zhang J. Y. Ma J. L. Li Z. X. Zhang L. Zhang Y. (2019). Effects of *Helicobacter pylori* treatment and vitamin and garlic supplementation on gastric cancer incidence and mortality: Follow-up of a randomized intervention trial. BMJ 366, l5016. 10.1136/bmj.l5016 31511230 PMC6737461

[B78] Liang D. Wu H. Wong M. W. Huang D. (2015). Diallyl trisulfide is a fast H2S donor, but diallyl disulfide is a slow one: The reaction pathways and intermediates of glutathione with polysulfides. Org. Lett. 17, 4196–4199. 10.1021/acs.orglett.5b01962 26301500

[B79] Liao Q. J. Su J. Zhou X. T. Tang H. L. Song Y. Su Q. (2007). Inhibitory effect of diallyl disulfide on proliferation of human colon cancer cell line SW480 in nude mice. Ai Zheng 26, 828–832. 17697541

[B80] Ling H. He J. Tan H. Yi L. Liu F. Ji X. (2017). Identification of potential targets for differentiation in human leukemia cells induced by diallyl disulfide. Int. J. Oncol. 50, 697–707. 10.3892/ijo.2017.3839 28101575

[B81] Ling H. Ji X. Lei Y. Jia Y. Liu F. Xia H. (2020). Diallyl disulfide induces downregulation and inactivation of cofilin 1 differentiation via the Rac1/ROCK1/LIMK1 pathway in leukemia cells. Int. J. Oncol. 56, 772–782. 10.3892/ijo.2020.4968 32124958 PMC7010219

[B82] Ling H. Lu L. F. He J. Xiao G. H. Jiang H. Su Q. (2014). Diallyl disulfide selectively causes checkpoint kinase-1 mediated G2/M arrest in human MGC803 gastric cancer cell line. Oncol. Rep. 32, 2274–2282. 10.3892/or.2014.3417 25176258

[B83] Ling H. Wen L. Ji X. X. Tang Y. L. He J. Tan H. (2010). Growth inhibitory effect and Chk1-dependent signaling involved in G 2/M arrest on human gastric cancer cells induced by diallyl disulfide. Braz. J. Med. Biol. Res. 43, 271–278. 10.1590/S0100-879X2010007500004 20401435

[B84] Liu R. Yang Y. N. Yi L. Qing J. Li Q. Y. Wang W. S. (2018). Diallyl disulfide effect on the invasion and migration ability of HL-60 cells with a high expression of DJ-1 in the nucleus through the suppression of the src signaling pathway. Oncol. Lett. 15, 6377–6385. 10.3892/ol.2018.8139 29725397 PMC5920463

[B85] Lu H. F. Yang J. S. Lin Y. T. Tan T. W. Ip S. W. Li Y. C. (2007). Diallyl disulfide induced signal transducer and activator of transcription 1 expression in human colon cancer colo 205 cells using differential display RT-PCR. Cancer Genomics Proteomics 4, 93–97. 17804871

[B86] Ma J. L. Zhang L. Brown L. M. Li J. Y. Shen L. Pan K. F. (2012). Fifteen-year effects of helicobacter pylori, garlic, and vitamin treatments on gastric cancer incidence and mortality. J. Natl. Cancer Inst. 104, 488–492. 10.1093/jnci/djs003 22271764 PMC3309129

[B87] Marie Hardwick J. Soane L. (2013). Multiple functions of BCL-2 family proteins. Cold Spring Harb. Perspect. Biol. 5, a008722. 10.1101/cshperspect.a008722 23378584 PMC3552500

[B88] Mikaili P. Maadirad S. Moloudizargari M. Aghajanshakeri S. Sarahroodi S. (2013). Therapeutic uses and pharmacological properties of garlic, shallot, and their biologically active compounds. Iran. J. Basic Med. Sci. 16, 1031–1048. 10.22038/ijbms.2013.1865 24379960 PMC3874089

[B89] Mitra S. Chakraborty A. J. Tareq A. M. Emran T. B. Nainu F. Khusro A. (2022a). Impact of heavy metals on the environment and human health: Novel therapeutic insights to counter the toxicity. J. King Saud Univ. - Sci. 34, 101865. 10.1016/j.jksus.2022.101865

[B90] Mitra S. Lami M. S. Ghosh A. Das R. Tallei T. E. Fatimawali (2022b). Hormonal therapy for gynecological cancers: How far has science progressed toward clinical applications? Cancers (Basel) 14, 759. 10.3390/cancers14030759 35159024 PMC8833573

[B91] Mitra S. Lami M. S. Uddin T. M. Das R. Islam F. Anjum J. (2022c). Prospective multifunctional roles and pharmacological potential of dietary flavonoid narirutin. Biomed. Pharmacother. 150, 112932. 10.1016/j.biopha.2022.112932 35413599

[B92] Mitra S. Rauf A. Tareq A. M. Jahan S. Emran T. B. Shahriar T. G. (2021). Potential health benefits of carotenoid lutein: An updated review. Food Chem. Toxicol. 154, 112328. 10.1016/j.fct.2021.112328 34111488

[B93] Mitra S. Sarker J. Mojumder A. Shibbir T. B. Das R. Emran T. B. (2022d). Genome editing and cancer: How far has research moved forward on CRISPR/Cas9? Biomed. Pharmacother. 150, 113011. 10.1016/j.biopha.2022.113011 35483191

[B94] Mitra S. Tareq A. M. Das R. Emran T. B. Nainu F. Chakraborty A. J. (2022e). Polyphenols: A first evidence in the synergism and bioactivities. Food Rev. Int., 1–23. 10.1080/87559129.2022.2026376

[B95] Moding E. J. Kastan M. B. Kirsch D. G. (2013). Strategies for optimizing the response of cancer and normal tissues to radiation. Nat. Rev. Drug Discov. 12, 526–542. 10.1038/nrd4003 23812271 PMC3906736

[B96] Munari C. C. De Oliveira P. F. Campos J. C. L. Martins S. D. P. L. Da Costa J. C. Bastos J. K. (2014). Antiproliferative activity of Solanum lycocarpum alkaloidic extract and their constituents, solamargine and solasonine, in tumor cell lines. J. Nat. Med. 68, 236–241. 10.1007/s11418-013-0757-0 23475509

[B97] Nkrumah-Elie Y. M. Reuben J. S. Hudson A. M. Taka E. Badisa R. Ardley T. (2012). The attenuation of early benzo(a)pyrene-induced carcinogenic insults by diallyl disulfide (DADS) in MCF-10A cells. Nutr. Cancer 64, 1112–1121. 10.1080/01635581.2012.712738 23006051 PMC3559020

[B98] Nussbaumer S. Bonnabry P. Veuthey J. L. Fleury-Souverain S. (2011). Analysis of anticancer drugs: A review. Talanta 85, 2265–2289. 10.1016/j.talanta.2011.08.034 21962644

[B99] Odewumi C. Latinwo L. M. Badisa V. L. Smith S. Cobb Abdullah A. Kent First M. (2019). Modulation of cadmium induced apoptotic, cancer and inflammation related cytokines by diallyl disulfide in rat liver cells. Ann. Toxicol. 1. 10.36959/736/633

[B100] Odom R. Y. Dansby M. Y. Rollins-Hairston A. M. Jackson K. M. Kirlin W. G. (2009). Phytochemical induction of cell cycle arrest by glutathione oxidation and reversal by N-acetylcysteine in human colon carcinoma cells. Nutr. Cancer 61, 332–339. 10.1080/01635580802549982 19373606 PMC2749979

[B101] Omar S. H. Al-Wabel N. A. (2010). Organosulfur compounds and possible mechanism of garlic in cancer. Saudi Pharm. J. 18, 51–58. 10.1016/j.jsps.2009.12.007 23960721 PMC3731019

[B102] Park H. S. Kim G. Y. Choi I. W. Kim N. D. Hwang H. J. Choi Y. W. (2011). Inhibition of matrix metalloproteinase activities and tightening of tight junctions by diallyl disulfide in AGS human gastric carcinoma cells. J. Food Sci. 76, T105–T111. 10.1111/j.1750-3841.2011.02114.x 22417372

[B103] Pinkerton R. (2013). “Non-Hodgkin lymphoma,” in Evidence-based pediatr. Oncol. Third Ed., 88–104. 10.1002/9781118625309.ch10

[B104] Rahman M. M. Islam F. Afsana Mim S. Khan M. S. Islam M. R. Haque M. A. (2022). Multifunctional therapeutic approach of nanomedicines against inflammation in cancer and aging. J. Nanomater., 1–19. 10.1155/2022/4217529

[B105] Ramirez D. A. Locatelli D. A. González R. E. Cavagnaro P. F. Camargo A. B. (2017). Analytical methods for bioactive sulfur compounds in Allium: An integrated review and future directions. J. Food Compost. Anal. 61, 4–19. 10.1016/j.jfca.2016.09.012

[B106] Rastegari F. Rafieian-Kopaei M. (2016). Antioxidant supplements and cancer. Immunopathol. Persa 2.

[B107] Rauf A. Abu-Izneid T. Khalil A. A. Imran M. Shah Z. A. Bin Emran T. (2021). Berberine as a potential anticancer agent: A comprehensive review. Molecules 26, 7368. 10.3390/molecules26237368 34885950 PMC8658774

[B108] Rauf A. Shariati M. A. Imran M. Bashir K. Khan S. A. Mitra S. (2022). Comprehensive review on naringenin and naringin polyphenols as a potent anticancer agent. Environ. Sci. Pollut. Res. Int. 29, 31025–31041. 10.1007/s11356-022-18754-6 35119637

[B109] Redza-Dutordoir M. Averill-Bates D. A. (2016). Activation of apoptosis signalling pathways by reactive oxygen species. Biochim. Biophys. Acta 1863, 2977–2992. 10.1016/j.bbamcr.2016.09.012 27646922

[B110] Rivlin R. S. (2009). Can garlic reduce risk of cancer? Am. J. Clin. Nutr. 89, 17–18. 10.3945/ajcn.2008.27181 19056577 PMC2647709

[B111] Sadjadi A. Marjani H. Semnani S. Nasseri-Moghaddam S. (2010). Esophageal cancer in Iran: A review. Middle East J. Cancer 1, 5–14.

[B112] Sánchez A. R. Fernández B. C. Raposo C. G. Castellanos P. C. (2013). Ovarian cancer. Med. - Programa Form. Médica Contin. Acreditado 11, 1641–1648. 10.1016/S0304-5412(13)70518-3

[B113] Saraf A. Dubey N. Dubey N. Sharma M. (2021). Enhancement of cytotoxicty of diallyl disulfide toward colon cancer by Eudragit S100/PLGA nanoparticles. J. Drug Deliv. Sci. Technol. 64, 102580. 10.1016/j.jddst.2021.102580

[B114] Saud S. M. Li W. Gray Z. Matter M. S. Colburn N. H. Young M. R. (2016). Diallyl disulfide (DADS), a constituent of garlic, inactivates NF-κB and prevents colitis-induced colorectal cancer by inhibiting GSK-3β. Cancer Prev. Res. 9, 607–615. 10.1158/1940-6207.CAPR-16-0044 PMC4930730 27138790

[B115] Shamshirian A. Alizadeh-Navaei R. Shamshirian A. Hedayatizadeh-Omran A. Ghadimi R. Janbabai G. (2018). Effect of garlic in gastric cancer prognosis: a systematic review and meta-analysis. WCRJ 5 (4), e1184.

[B116] Shan Y. Wei Z. Tao L. Wang S. Zhang F. Shen C. (2016). Prophylaxis of diallyl disulfide on skin carcinogenic model via p21-dependent Nrf2 stabilization. Sci. Rep. 6, 35676. 10.1038/srep35676 27759091 PMC5069634

[B117] Shin D. Y. Kim G. Y. Kim J. I. Yoon M. K. Kwon T. K. Lee S. J. (2010). Anti-invasive activity of diallyl disulfide through tightening of tight junctions and inhibition of matrix metalloproteinase activities in LNCaP prostate cancer cells. Toxicol. Vitro. 24, 1569–1576. 10.1016/j.tiv.2010.06.014 20600798

[B118] Shin D. Y. Kim G. Y. Lee J. H. Choi B. T. Yoo Y. H. Choi Y. H. (2012). Apoptosis induction of human prostate carcinoma DU145 cells by diallyl disulfide via modulation of JNK and PI3K/AKT signaling pathways. Int. J. Mol. Sci. 13, 14158–14171. 10.3390/ijms131114158 23203057 PMC3509573

[B119] Siddhartha V. T. Pindiprolu S. K. S. S. Chintamaneni P. K. Tummala S. Nandha Kumar S. (2018). RAGE receptor targeted bioconjuguate lipid nanoparticles of diallyl disulfide for improved apoptotic activity in triple negative breast cancer: *In vitro* studies. Artif. Cells Nanomed. Biotechnol. 46, 387–397. 10.1080/21691401.2017.1313267 28415882

[B120] Siegel R. L. Miller K. D. Jemal A. (2016). Cancer statistics, 2016. Ca. Cancer J. Clin. 66, 7–30. 10.3322/caac.21332 26742998

[B121] Siegel R. L. Miller K. D. Jemal A. (2018). Cancer statistics, 2018. Ca. Cancer J. Clin. 68, 7–30. 10.3322/caac.21442 29313949

[B122] Society A. C. (2016). Cancer Facts & Figures 2016, 2016, 1–9.Cancer Facts Fig.

[B123] Song J. D. Lee S. K. Kim K. M. Park S. E. Park S. J. Kim K. H. (2009). Molecular mechanism of diallyl disulfide in cell cycle arrest and apoptosis in HCT-116 colon cancer cells. J. Biochem. Mol. Toxicol. 23, 71–79. 10.1002/jbt.20266 19202565

[B124] Song X. Yue Z. Nie L. Zhao P. Zhu K. Wang Q. (2021). Biological functions of diallyl disulfide, a garlic-derived natural organic sulfur compound. Evid. Based. Complement. Altern. Med. 2021, 5103626. 10.1155/2021/5103626 PMC8570849 34745287

[B125] Su B. (2012). Diallyl disulfide increases histone acetylation and P21WAF1 expression in human gastric cancer cells *in vivo* and *in vitro* . Biochem. Pharmacol. 01. 10.4172/2167-0501.1000106

[B126] Su B. Jian S. U. Zeng Y. Ding E. Liu F. Tan T. (2018). Diallyl disulfide inhibits TGF-β1-induced upregulation of Rac1 and β-catenin in epithelial-mesenchymal transition and tumor growth of gastric cancer. Oncol. Rep. 39, 2797–2806. 10.3892/or.2018.6345 29620286

[B127] Su B. Su J. He H. Wu Y. Xia H. Zeng X. (2015). Identification of potential targets for diallyl disulfide in human gastric cancer MGC-803 cells using proteomics approaches. Oncol. Rep. 33, 2484–2494. 10.3892/or.2015.3859 25812569

[B128] Su J. Zhou Y. Pan Z. Shi L. Yang J. Liao A. (2017). Downregulation of LIMK1–ADF/cofilin by DADS inhibits the migration and invasion of colon cancer. Sci. Rep. 7, 45624. 10.1038/srep45624 28358024 PMC5372356

[B129] Suangtamai T. Tanyong D. I. (2016). Diallyl disulfide induces apoptosis and autophagy via mTOR pathway in myeloid leukemic cell line. Tumour Biol. 37, 10993–10999. 10.1007/s13277-016-4989-y 26891668

[B130] Sujatha P. Anantharaju P. G. Veeresh P. M. Dey S. Bovilla V. R. Madhunapantula S. V. (2017). Diallyl disulfide (DADS) retards the growth of breast cancer cells *in vitro* and *in vivo* through apoptosis induction. Biomed. Pharmacol. J. 10, 1619–1630. 10.13005/bpj/1273

[B131] Sun J. Mu H. Yu J. Li L. Yan H. Li G. (2019). Diallyl disulfide down-regulates calreticulin and promotes C/EBPα expression in differentiation of human leukaemia cells. J. Cell. Mol. Med. 23, 194–204. 10.1111/jcmm.13904 30394654 PMC6307788

[B132] Sung H. Ferlay J. Siegel R. L. Laversanne M. Soerjomataram I. Jemal A. (2021). Global cancer statistics 2020: GLOBOCAN estimates of incidence and mortality worldwide for 36 cancers in 185 countries. Ca. Cancer J. Clin. 71, 209–249. 10.3322/caac.21660 33538338

[B133] Sung H. Ferlay J. Siegel R. L., (2020). GLOBOCAN estimates of incidence and mortality worldwide for 36 cancers in 185 countries. Glob. Cancer Stat. 71. 10.3322/caac.21660 33538338

[B134] Tait S. W. G. Green D. R. (2010). Mitochondria and cell death: Outer membrane permeabilization and beyond. Nat. Rev. Mol. Cell Biol. 11, 621–632. 10.1038/nrm2952 20683470

[B135] Talluri S. V. Kuppusamy G. Karri V. V. S. R. Yamjala K. Wadhwani A. Madhunapantula S. R. V. (2017). Application of quality-by-design approach to optimize diallyl disulfide-loaded solid lipid nanoparticles. Artif. Cells Nanomed. Biotechnol. 45, 474–488. 10.3109/21691401.2016.1173046 27112220

[B136] Tan H. Xiao-xia W. J. (2011). Growth inhibition and apoptosis of K562 cells induced by diallyl disulfide. South China,Hengyang,Hunan 421001,China: Inst. Cancer Res.

[B137] Tan L. Zhang M. Xia L. Mei H. Z. Zhi Y. Li J. (2004). The initiation of G2/M checkpoint by diallyl disulfide requires the activation of p38 MAP kinase in HL-60 cells. Zhonghua Xue Ye Xue Za Zhi 25, 273–276. 15182534

[B138] Tang H. Kong Y. Guo J. Tang Y. Xie X. Yang L. (2013). Diallyl disulfide suppresses proliferation and induces apoptosis in human gastric cancer through Wnt-1 signaling pathway by up-regulation of miR-200b and miR-22. Cancer Lett. 340, 72–81. 10.1016/j.canlet.2013.06.027 23851184

[B139] Thompson A. K. (2014). Health-promoting properties of fruit and vegetables. Fruit. Veg., 557–572. 10.1002/9781118653975.ch14

[B140] Tsubura A. Lai Y.-C. Kuwata M. Uehara N. Yoshizawa K. (2012). Anticancer effects of garlic and garlic-derived compounds for breast cancer control. Anticancer. Agents Med. Chem. 11, 249–253. 10.2174/187152011795347441 21269259

[B141] UICC (2020). Globocan 2020: New global cancer data, 5. New York, USA: Website.

[B142] Van Den Heuvel-Eibrink M. (2004). “Acute myeloid leukaemia,” in Paediatr. Oncol. Third Ed., 203–229. 10.1201/b13276-14

[B143] Wang X. Jiao F. Wang Q. W. Wang J. Yang K. Hu R. R. (2012). Aged black garlic extract induces inhibition of gastric cancer cell growth *in vitro* and *in vivo* . Mol. Med. Rep. 5, 66–72. 10.3892/mmr.2011.588 21922142

[B144] Wang Y. Zhang L. Moslehi R. Ma J. Pan K. Zhou T. (2009). Long-term garlic or micronutrient supplementation, but not anti-Helicobacter pylori therapy, increases serum folate or glutathione without affecting serum vitamin B-12or homocysteineina rural Chinese population. J. Nutr. 139, 106–112. 10.3945/jn.108.091389 19056661 PMC2646216

[B145] Wei Z. Shan Y. Tao L. Liu Y. Zhu Z. Liu Z. (2017). Diallyl trisulfides, a natural histone deacetylase inhibitor, attenuate HIF-1α synthesis, and decreases breast cancer metastasis. Mol. Carcinog. 56, 2317–2331. 10.1002/mc.22686 28574600

[B146] Williams M. M. Lee L. Werfel T. Joly M. M. M. Hicks D. J. Rahman B. (2018). Intrinsic apoptotic pathway activation increases response to anti-estrogens in luminal breast cancers. Cell Death Dis. 9, 21. 10.1038/s41419-017-0072-x 29343814 PMC5833697

[B147] Wu P. P. Chung H. W. Liu K. C. Wu R. S. C. Yang J. S. Tang N. Y. (2011). Diallyl sulfide induces cell cycle arrest and apoptosis in HeLa human cervical cancer cells through the p53, caspase- and mitochondria-dependent pathways. Int. J. Oncol. 38, 1605–1613. 10.3892/ijo.2011.973 21424116

[B148] Wu X. Shi J. Fang W. xia Guo, X. Zhang L. Yun L (2019). Allium vegetables are associated with reduced risk of colorectal cancer: A hospital-based matched case-control study in China. Asia. Pac. J. Clin. Oncol. 15, e132–e141. 10.1111/ajco.13133 30790463

[B149] Xia L. Lin J. Su J. Oyang L. Wang H. Tan S. (2019). Diallyl disulfide inhibits colon cancer metastasis by suppressing Rac1-mediated epithelial-mesenchymal transition. Onco. Targets. Ther. 12, 5713–5728. 10.2147/OTT.S208738 31410018 PMC6645609

[B150] Xiao X. Chen B. Liu X. Liu P. Zheng G. Ye F. (2014). Diallyl disulfide suppresses SRC/Ras/ERK signaling-mediated proliferation and metastasis in human breast cancer by up-regulating miR-34a. PLoS One 9, e112720. 10.1371/journal.pone.0112720 25396727 PMC4232521

[B151] Xie X. Huang X. Tang H. Ye F. Yang L. Guo X. (2018). Diallyl disulfide inhibits breast cancer stem cell progression and glucose metabolism by targeting CD44/PKM2/AMPK signaling. Curr. Cancer Drug Targets 18, 592–599. 10.2174/1568009617666171024165657 29110616

[B152] Xu S. Huang H. Tang D. Xing M. Zhao Q. Li J. (2021). Diallyl disulfide attenuates ionizing radiation-induced migration and invasion by suppressing Nrf2 signaling in non–small-cell lung cancer. Dose. Response. 19, 15593258211033114. 10.1177/15593258211033114 34393685 PMC8351038

[B153] Yamaguchi Y. Kumagai H. (2019). Characteristics, biosynthesis, decomposition, metabolism and functions of the garlic odour precursor, S-allyl-l-cysteine sulfoxide (Review). Exp. Ther. Med. 10.3892/etm.2019.8385 PMC6966203 32010334

[B154] Yang J. Liu X. Cao S. Dong X. Rao S. Cai K. (2020). Understanding esophageal cancer: The challenges and opportunities for the next decade. Front. Oncol. 10, 1727. 10.3389/fonc.2020.01727 33014854 PMC7511760

[B155] Yang J. S. Chen G. W. Hsia T. C. Ho H. C. Ho C. C. Lin M. W. (2009). Diallyl disulfide induces apoptosis in human colon cancer cell line (COLO 205) through the induction of reactive oxygen species, endoplasmic reticulum stress, caspases casade and mitochondrial-dependent pathways. Food Chem. Toxicol. 47, 171–179. 10.1016/j.fct.2008.10.032 19038304

[B156] Ye X. Yin X. (2017). Effect of MEK-ERK signaling pathway on diallyl disulfide-induced autophagv in human leukemia K562 cells. J. Leuk. Lymphoma 26, 665–669. 10.3760/cma.j.issn.1009-9921.2017.11.007

[B157] Yi L. Ji X. X. Tan H. Lin M. Tang Y. Wen L. (2010). Role of Ras-related C3 botulinum toxin substrate 2 (Rac2), NADPH oxidase and reactive oxygen species in diallyl disulphide-induced apoptosis of human leukaemia HL-60 cells. Clin. Exp. Pharmacol. Physiol. 37, 1147–1153. 10.1111/j.1440-1681.2010.05444.x 20804509

[B158] Yi L. Shan J. Chen X. Li G. Li L. Tan H. (2016). Involvement of calreticulin in cell proliferation, invasion and differentiation in diallyl disulfide-treated HL-60 cells. Oncol. Lett. 12, 1861–1867. 10.3892/ol.2016.4850 27588133 PMC4998039

[B159] Yi L. Su Q. (2013). Molecular mechanisms for the anti-cancer effects of diallyl disulfide. Food Chem. Toxicol. 57, 362–370. 10.1016/j.fct.2013.04.001 23583486

[B160] Yin X. Feng C. Han L. Ma Y. Jiao Y. Wang J. (2018). Diallyl disulfide inhibits the metastasis of type Ⅱ esophageal‑gastric junction adenocarcinoma cells via NF-κB and PI3K/AKT signaling pathways *in vitro* . Oncol. Rep. 39, 784–794. 10.3892/or.2017.6113 29207122

[B161] Yin X. Zhang J. Li X. Liu D. Feng C. Liang R. (2014a). DADS suppresses human esophageal xenograft tumors through RAF/MEK/ERK and mitochondria-dependent pathways. Int. J. Mol. Sci. 15, 12422–12441. 10.3390/ijms150712422 25026173 PMC4139851

[B162] Yin X. Zhang R. Feng C. Zhang J. Liu D. Xu K. (2014b). Diallyl disulfide induces G2/M arrest and promotes apoptosis through the p53/p21 and MEK-ERK pathways in human esophageal squamous cell carcinoma. Oncol. Rep. 32, 1748–1756. 10.3892/or.2014.3361 25175641

[B163] You W. C. Brown L. M. Zhang L. Li J. Y. Jin M. L. Chang Y. S. (2006). Randomized double-blind factorial trial of three treatments to reduce the prevalence of precancerous gastric lesions. J. Natl. Cancer Inst. 98, 974–983. 10.1093/jnci/djj264 16849680

[B164] Yu F. S. Yu C. S. Lin J. P. Chen S. C. Lai W. W. Chung J. G. (2005). Diallyl disulfide inhibits N-acetyltransferase activity and gene expression in human esophagus epidermoid carcinoma CE 81T/VGH cells. Food Chem. Toxicol. 43, 1029–1036. 10.1016/j.fct.2005.02.009 15833378

[B165] Zhang N. Wang Y. Zhang J. Liu B. Li G. Xin S. (2019a). Diallyl disulfide attenuates non‑alcoholic steatohepatitis by suppressing key regulators of lipid metabolism, lipid peroxidation and inflammation in mice. Mol. Med. Rep. 20, 1363–1372. 10.3892/mmr.2019.10316 31173200

[B166] Zhang R. Shi H. Ren F. Liu Z. Ji P. (2019b). Effects of diallyl disulfide on the proliferation and apoptosis of epithelial ovarian cancer cells by inducing G2/M arrest. Zhongguo Yi Xue Ke Xue Yuan Xue Bao. 41, 43–52. 10.3881/j.issn.1000-503X.10494 30837041

[B167] Zhang Y. Liu X. Ruan J. Zhuang X. Zhang X. Li Z. (2020). Phytochemicals of garlic: Promising candidates for cancer therapy. Biomed. Pharmacother. 123, 109730. 10.1016/j.biopha.2019.109730 31877551

[B168] Zhou Y. Li Y. Zhou T. Zheng J. Li S. Li H. B. (2016). Dietary natural products for prevention and treatment of liver cancer. Nutrients 8, 156. 10.3390/nu8030156 26978396 PMC4808884

[B169] Zhou Z. Tang M. Liu Y. Zhang Z. Lu R. Lu J. (2017). Apigenin inhibits cell proliferation, migration, and invasion by targeting Akt in the A549 human lung cancer cell line. Anticancer. Drugs 28, 446–456. 10.1097/CAD.0000000000000479 28125432

[B170] Zuniga K. E. Parma D. L. Muñoz E. Spaniol M. Wargovich M. Ramirez A. G. (2019). Dietary intervention among breast cancer survivors increased adherence to a mediterranean-style, anti-inflammatory dietary pattern: The rx for better breast health randomized controlled trial. Breast Cancer Res. Treat. 173, 145–154. 10.1007/s10549-018-4982-9 30259284 PMC6387648

